# Evolving contact mechanics and microstructure formation dynamics of the lithium metal-Li_7_La_3_Zr_2_O_12_ interface

**DOI:** 10.1038/s41467-021-26632-x

**Published:** 2021-11-04

**Authors:** Wesley Chang, Richard May, Michael Wang, Gunnar Thorsteinsson, Jeff Sakamoto, Lauren Marbella, Daniel Steingart

**Affiliations:** 1 Department of Mechanical and Aerospace Engineering, Princeton University, Princeton, NJ 08544 USA; 2 Andlinger Center for Energy and the Environment, Princeton University, Princeton, NJ 08544 USA; 3 Department of Chemical Engineering, Columbia University, New York, NY 10027 USA; 4 Columbia Electrochemical Energy Center, Columbia University, New York, NY 10027 USA; 5 Department of Materials Science and Engineering, University of Michigan, Ann Arbor, MI 48104 USA; 6 Department of Earth and Environmental Engineering, Columbia University, New York, NY 10027 USA; 7 Department of Mechanical Engineering, University of Michigan, Ann Arbor, MI 48104 USA

**Keywords:** Batteries, Characterization and analytical techniques, Solid-state NMR, Energy

## Abstract

The dynamic behavior of the interface between the lithium metal electrode and a solid-state electrolyte plays a critical role in all-solid-state battery performance. The evolution of this interface throughout cycling involves multiscale mechanical and chemical heterogeneity at the micro- and nano-scale. These features are dependent on operating conditions such as current density and stack pressure. Here we report the coupling of operando acoustic transmission measurements with nuclear magnetic resonance spectroscopy and magnetic resonance imaging to correlate changes in interfacial mechanics (such as contact loss and crack formation) with the growth of lithium microstructures during cell cycling. Together, the techniques reveal the chemo-mechanical behavior that governs lithium metal and Li_7_La_3_Zr_2_O_12_ interfacial dynamics at various stack pressure regimes and with voltage polarization.

## Abstract (web summary)

All-solid-state batteries are promising alternatives to Li-ion batteries. Here, the authors investigate the chemo-mechanical changes at the lithium metal/solid electrolyte interface via operando acoustic transmission and magnetic resonance imaging.

## Introduction

Solid-state electrolytes for high energy density lithium metal batteries exhibit a variety of improved electrochemical and physical properties compared to their liquid-state counterparts^1^. The Li_7_La_3_Zr_2_O_12_ (garnet-type LLZO) solid-state electrolytes have ionic conductivities comparable to liquid electrolytes^2^, and are electrochemically stable up to ~3 V vs Li^+^/Li^3,4^. However, further optimization of the Li (s)–LLZO interface is required because lithium propagation through the LLZO electrolyte presents major issues at practical current densities^5,6^. Optimization of this interface is challenging because the transient chemical and mechanical mechanisms responsible for lithium microstructural growth in solid electrolytes are not readily observed, especially during battery operation. Apart from lithium filament protrusion on the plating side, prior work has also suggested that lithium stripping initiates a void-forming failure mode^7,8^. Poor interfacial contact from voids during stripping (discharge) leads to rate limitations, whereby lithium diffusion and creep towards the interface is unable to keep up with lithium depletion and migration into the electrolyte^7^. The formation of voids, or gaps between the stripping electrode and the solid electrolyte, leads to an increase in the interfacial resistance and an increase in the measured cell voltage, which we define as voltage polarization^7^.

These hypotheses have primarily been supported by electrochemical analyses and post-mortem optical or microscopic observations^9–11^. For example, Sharafi et al.^10^ characterized the Li (s)–LLZO interfacial kinetics using electrochemical impedance spectroscopy (EIS) to measure the various contributions to cell impedance as a function of temperature. Cheng et al.^6^ imaged surface cracks with scanning electron microscopy (SEM) and subsurface cracks with focused-ion-beam milling (FIB-SEM), along with surface chemical analysis with energy dispersive X-ray spectroscopy (EDS). These ex situ characterization techniques require cell disassembly for microscopy or spectroscopy, and provide no information on cell behavior during operation. The complex evolution of the Li (s)–LLZO interface during lithium plating and lithium stripping further benefits from operando characterization for an enhanced mechanistic understanding of coupled contact mechanics and microstructure formation heterogeneity.

By observing the cell changes during operation (i.e., during galvanostatic plating and stripping), operando characterization techniques can unveil dynamic interactions within the cell more clearly than post-mortem analysis^12^. Yet, operando characterization of the Li (s)–LLZO interface is challenging because of the inherent difficulties associated with probing buried solid–solid interfaces at the relevant length and timescales. In particular, synchrotron X-ray tomography provides high spatial resolution down to less than 50 μm, but X-ray resolution is directly related to elemental mass^13^. Lithium filaments and voids are, therefore, both X-ray transparent. Operando optical microscopy has been recently developed by Kazyak et al.^14^ utilizing a custom-designed cell with an optical viewing plane, which brings further visual insight to the evolving dynamics but also has a different cell geometry than the present study. Operando neutron depth profiling and nuclear magnetic resonance (NMR) both overcome the low sensitivity of lithium in the case of X-ray-based operando methods, by probing neutrons and nuclei, respectively^15–18^. Various operando characterization techniques for solid-state batteries have been well summarized in recent reviews by Strauss et al., Lou et al., and Tan et al.^12^^,^^19^^,^^20^.

In this work, we combine operando acoustic, NMR, and magnetic resonance imaging (MRI) techniques to probe the coupled relationship between interface mechanics, interphase chemistry, and microstructural formation at the mesoscale and microscale. Operando acoustic transmission can detect changes in cell modulus, density, and expansion in conventional lithium-ion cells^21,22^. Acoustic transmission utilizes sound waves at ultrasound frequencies (typically between 1 and 10 MHz) which propagate through the cell layers, with sound speed and amplitude dependent on the mechanical properties of each layer^23^. Since solid/gas interfaces have poor acoustic signal transmission due to a high acoustic impedance mismatch, even minor contact loss at the stripped Li (s)–LLZO interface is expected to attenuate the acoustic amplitude^24^. In the presence of a solid/gas interface, propagating sound waves are mostly reflected back. Unlike conventional lithium-ion batteries with liquid electrolytes and phase changing graphite negative electrodes, lithium metal negative electrodes paired with solid-state electrolytes present a different medium for sound propagation. Instead of amplitude and sound speed changes due to bulk phase change, lithium plating and stripping increases the interfacial roughness between the lithium metal and the electrolyte. Contact loss due to lithium stripping forming voids decreases the intensity of the propagated wave, resulting in a loss of sound transmission intensity. This stochastic effect of interfacial roughness has been studied experimentally and computationally in other fields, but the effect of electrochemically induced nucleation, deposition, and stripping on acoustic transmission has not been thoroughly investigated^25,26^. Further explanation of the impact of interfacial roughness on acoustic wave transmission is provided in Supplementary Discussion 1.1 and Supplementary Fig. 1.

**Fig. 1:**
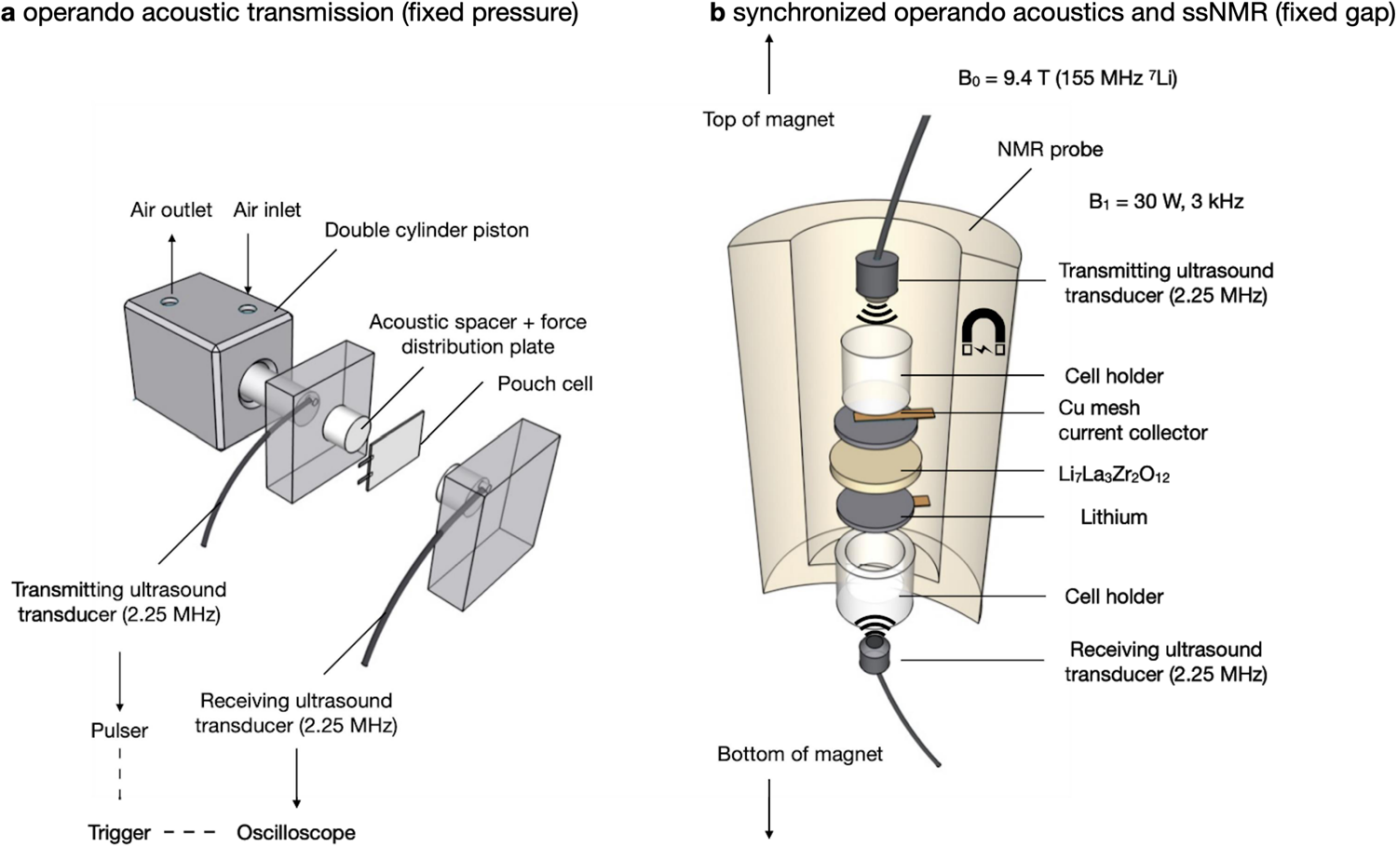
Schematic of experimental designs and cell fixtures. **a** Fixed pressure configuration for operando acoustic transmission experiments, utilizing a double-piston pneumatic cylinder for precise control of stack pressure. **b** Fixed gap configuration for synchronized operando acoustic transmission and ssNMR spectroscopy. Acoustic transmission probes changes in interfacial contact mechanics and ssNMR measures the rate of microstructure formation.

Solid-state nuclear magnetic resonance (ssNMR) and MRI complement the acoustic measurements by simultaneously probing chemical composition and microstructure morphology/quantity^17,27^. We exploit this unique chemo-mechanical probe to evaluate the minimum stack pressure required to maintain conformal contact and interfacial stability (or lack thereof) over long voltage polarization steps. In particular, we apply stack pressures from 2 up to 13 MPa during lithium plating and stripping of symmetric Li (s)–LLZO cells to measure the effect of increasing stack pressure on void formation and creep recovery rate with acoustic transmission (Fig. 1a). The lower stack pressure range studied (between 2 and 4 MPa) is consistent with other LLZO studies^7^, so we explore a further increase in stack pressure to measure the effect of lithium plastic deformation. The higher stack pressure of 13 MPa is near the theoretically determined value by Zhang et al.^28^ where full contact of lithium metal with the solid electrolyte was predicted. Our experiments indicate that a stack pressure above a critical threshold during rest reversibly recovers contact loss on the stripped lithium side. The rates of contact loss at stack pressures of 2, 7.4, and 13 MPa are then correlated with the degree of lithium microstructural growth throughout the cell as detected by MRI. Lithium “microstructure” used herein refers to any non-planar form of lithium growth morphology at the Li (s)–LLZO interface and within the electrolyte, which encompasses interfacial voids and growth penetration through the solid-state electrolyte^29–31^. The combination of acoustics followed by ex situ MRI furthers understanding of the Li (s)–LLZO interface, because it correlates contact extent due to lithium stripping with lithium growth morphology near the buried interface. Besides the fixed pressure studies, we also design a fixed gap cell (Fig. 1b) for conducting synchronized operando characterization of both acoustic transmission and ssNMR spectroscopy (rather than post-mortem ^7^Li MRI imaging, which provides spatially resolved information but with lower temporal resolution). This capability is useful for characterizing the mechanics and microstructure for the same cell under operation, without noise from cell-to-cell variations.

## Results and discussion

### Electrochemical behavior of symmetric lithium metal–LLZO cells

The garnet LLZO (composition Li_7_La_3_Zr_2_O_12_) electrolyte synthesized from the well-characterized rapid induction hot-press technique is used as a model system (further details in the “Methods” section)^32^. The resulting LLZO electrolyte pellets have been verified in a previous report for chemical stability, reproducibility, and low defect surfaces from intensive polishing protocols^32^. For Li/LLZO/Li symmetric cells, the typical galvanostatic charge current used in this study was 0.2 mA/cm^2^, lower than the critical current density (CCD) of 0.6–0.7 mA/cm^2^ measured at 2 MPa stack pressure and the CCD of 1.4–1.5 mA/cm^2^ at 7 MPa stack pressure. These values were validated in the fixed pressure cell with current ramp tests (Supplementary Fig. 2). The CCD at a given stack pressure is an often-described figure-of-merit that indicates the current rates above which irreversible electrochemical shorting occurs^33^. At current densities higher than the CCD, the Li (s)–LLZO interface is known to be unstable and induce Griffith-like crack propagation as first introduced by De Jonghe et al.^34^ and adopted by Porz et al.^5^ for LLZO. At current densities below the CCD at the given stack pressure, no lithium penetration within the electrolyte is expected according to Sharafi et al.^10^ Since practical solid-state electrolytes are designed with a sufficient CCD to cycle stably at relevant current densities, the present work primarily explores interfacial dynamics at values below the CCD. A practical solid-state battery would involve cathode loadings of 3–4 mAh/cm^2^, implying a 1 C charge and a discharge current rate of 3–4 mA/cm^2^, and a requirement for the CCD to be at least 3–4 mA/cm^2^ to mitigate the chance of short circuiting.

Polarization tests were conducted at selected stack pressure regimes in a 30 °C chamber to explore the dynamic relationship between interfacial contact and microstructural growth at low current densities over a long duration of charge. The Li (s)–LLZO interface may be unstable at low stack pressures due to loss of interfacial contact from void formation on the stripped lithium foil side. When the rate of contact loss surpasses the rate of lithium creep, then the rise in overpotential due to loss of electrochemically active surface area may not recover^33^. Previously, Koshikawa et al.^35^ conducted three-electrode cell measurements and attributed cell impedance increase to void formation caused first by lithium dissolution, then exacerbated by subsequent lithium deposition. Wang et al.^7^ also observed cell resistance increase dominated by lithium stripping, defining a “critical stack pressure” which delineates stable stack pressure limits at a given current rate.

### Void-driven interfacial dynamics at 2 MPa

**Fig. 2:**
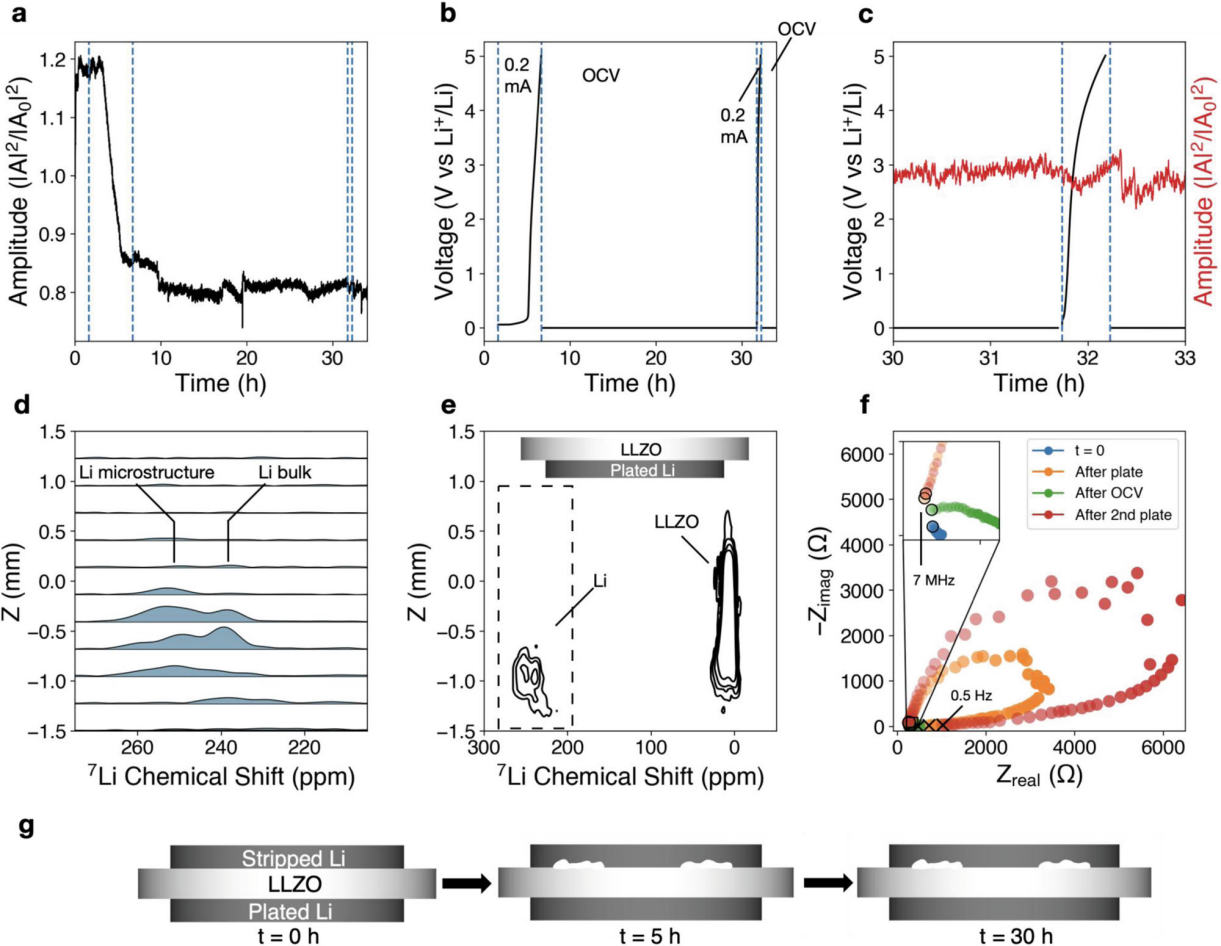
Lithium metal–LLZO interface at 2 MPa. Polarization test of Li (s)–LLZO interface at 2 MPa stack pressure conducted in a Li/LLZO/Li cell. After a few hours of initial mechanical equilibration, 0.2 mA/cm^2^ is applied galvanostatically until the voltage cutoff of 5 V, followed by a 25 h OCV rest step, a second polarization step in the same direction, and a second OCV rest step. **a** Normalized acoustic amplitude (intensities relative to first waveform intensity). **b** Voltage showing increase in overpotential to voltage cutoff during the first galvanostatic current step of 0.2 mA/cm^2^, and rapid overpotential increase during the second step. Vertical guidelines (dotted blue lines) are shown as a visual aid to delineate start and stop of galvanostatic current. **c** Voltage and amplitude curves zoomed in on the second polarization region. **d** 1D slices of the ex situ ^7^Li chemical shift imaging (CSI) experiment in the *z*-direction highlighting the lithium metal region between 200 and 280 ppm, as indicated by the dashed region in **e**. **e** Contour plot of ex situ ^7^Li CSI depicting a strong lithium metal resonance (~250 ppm) extending into the electrolyte region (0 ppm) in the *z*-direction. Stripped side is on top (positive *z*-direction) and the stripped foil at low stack pressures is missing because it immediately delaminates from the LLZO surface following disassembly. **f** Nyquist plot from potentiostatic EIS conducted during initial OCV, after the first galvanostatic step, after the subsequent 25 h OCV hold, and after the second galvanostatic step, indicating an increase in cell impedance following the polarization steps and incomplete impedance recovery following the 25 h OCV step. The open circle indicates the high-frequency start point (7 MHz), and the cross indicates the low-frequency end point (0.5 Hz). **g** Simplified cartoon illustration of interface behavior (showing extent of contact loss and presence/absence of voids) implied by the experimental data; not drawn to scale.

Acoustic transmission is used to measure interfacial mechanical changes at the mesoscale in Li/LLZO/Li cells at 2 MPa stack pressure. A stack pressure of 2 MPa resulted in immediate acoustic amplitude attenuation after applying 0.2 mA/cm^2^ (Fig. 2a). This attenuation progresses until cell polarization after ~5 h, or ~1 mAh of charge passed (Fig. 2b). The amplitude attenuation rate here reflects non-linear and volume-dependent contact loss due to the nature of spherical sound waves, which are affected by not only the interfacial contact but also the direction and morphology of such contact. Over the course of the subsequent 25 h open-circuit-voltage (OCV) step, there is minimal amplitude recovery. This suggests that the interfacial changes are governed by void formation and interfacial contact loss at 2 MPa and 0.2 mA/cm^2^. The formation of a void space is akin to the appearance of a solid/gas interface, which has a high acoustic impedance and prevents sound waves from propagating through. A roughened interface will also cause acoustic wave scattering, decreasing the amplitude of the transmitted wave. The effect of creep-dominated movement is later shown when exploring higher stack pressure regimes. After the 25 h OCV recovery step, a second galvanostatic polarization test results in a rapid overpotential increase in less than 30 min (Fig. 2c). EIS confirmed the increase in cell impedance after cell polarization, and incomplete impedance recovery after the 25 h OCV hold, indicating that this stack pressure is insufficient for creep-driven interfacial recovery (see below). The initial total cell impedance at *t* = 0 h of 250 Ω (indicated by the blue curve) increases to around 3000 Ω after the polarization step (orange), decreases back to ~500 Ω after the rest step, and then increases to over 6000 Ω after the second polarization step. The abnormal behavior of the low-frequency regime where a decrease in the real impedance portion is observed is attributed to an unstable interface, such that the electrochemical interfacial area changes within the time constant of the impedance test.

Having explored bulk contact loss, we next used chemical shift imaging (CSI), a ^7^Li MRI technique, to measure lithium growth inside the electrolyte ex situ (Fig. 2d, e). Lithium microstructure from either voids, uneven plating, or growth within the electrolyte manifest as higher frequency ^7^Li chemical shifts, compared to the native lithium metal electrode, due to differences in orientation with respect to the external magnetic field (i.e., bulk magnetic susceptibility effects, further explained in Supplementary Discussion 1.2)^36^. ^7^Li CSI showed a strong microstructural signal at ~250 ppm (Fig. 2e, *x*-axis, distinct from the bulk Li electrode at ~235 ppm) near the Li (s)–LLZO interface that extends into the solid electrolyte (^7^Li shift centered at ~0 ppm) after the polarization and repolarization test at 2 MPa. The distance of microstructural growth in the electrolyte is indicated by the position of the lithium microstructure peak on the *y*-axis of Fig. 2e; individual slices from the CSI experiment are shown in Fig. 2d. The CSI results are consistent with rapid surface roughening, which appears despite the implementation of a long rest step. The increased surface roughening observed after polarization/repolarization is correlated with the acoustic amplitude attenuation without subsequent recovery. A similar or greater magnitude of surface roughening could be assumed for the stripped side, which easily delaminated from the cell following disassembly, likely due to extensive void formation at the stripped Li (s)–LLZO interface. Analysis of the plated lithium–LLZO interface with ^7^Li CSI shows new peaks at approximately −1 and 8 ppm (Supplementary Fig. 3a, b), suggesting that LLZO decomposes and forms  an electronically conductive interphase, which may have been caused by the voltage polarization and contribute to interfacial reactivity and poor impedance recovery between the first and second Li plating steps (Fig. 2f). This is supported by post-mortem EDS measurements that show a rough, but conformal, interface on the plated side along with increased oxygen content near the interface (Supplementary Fig. 3c, d), in contrast with the stripped side shown in Supplementary Fig. 3e, f. We caution that artifacts may occur due to destructive nature of SEM/EDS sample preparation (cell cross-sectioning), as opposed to non-destructive ^7^Li CSI where the disassembled cell is enclosed in a vial and never exposed to air. Additional analysis of the chemical state of the interface is discussed in Supplementary Discussion 1.3 and Supplementary Fig. 3. Analysis of the subtle changes in acoustic amplitude and OCV during the 25 h rest step (Supplementary Fig. 4) suggests that slight changes in amplitude are correlated with similar 10–20 μV changes in the OCV. A duplicate test cell at 2 MPa can also be viewed in Supplementary Fig. 5, with consistent results. The amplitude attenuates immediately after applying current, and recovers slightly during the 25 h OCV (Supplementary Fig. 5a–c). This is correlated with the appearance of a lithium microstructure peak in the ^7^Li CSI around 255 ppm (Supplementary Fig. 5d, e), and a higher cell impedance after the polarization and rest protocol (Supplementary Fig. 5f).

Wang et al.^7^ postulated that a sufficiently high stack pressure should drive lithium creep toward the interface resulting in minimal void formation. They observed, for 0.2 mA/cm^2^, minimal overpotential increase at stack pressures of 2 MPa and above (with 4 mAh/cm^2^ charge passed), some increase in overpotential at 1.6 MPa (with 5 mAh/cm^2^ charge passed), and a significant amount of overpotential increase at 1.2 MPa (6 mAh/cm^2^). Conversely, Zhang et al.^28^ presented a contact model taking into account factors other than stack pressure, including lithium elastoplasticity and creep, and found that at least 20 MPa is required for maintaining conformal contact. The assumed lithium metal yield strength of 14 MPa is substantially higher than the conventionally measured ~0.7–0.8 MPa for bulk lithium metal^37^, and is more in line with measurements conducted at the nanoscale by Greer et al.^38^. Results from our combined acoustic transmission and ^7^Li CSI analyses show that 2 MPa stack pressure results in an immediate loss of contact, supporting the hypothesis that the theoretical yield strength of ~0.7–0.8 MPa for bulk lithium is not an accurate indicator for interfacial contact stability (Fig. 2g depicts a simplified illustration). Localized asperities may have significantly different yield strengths than the average bulk yield strength and require higher stack pressures to induce creep.

### Creep-dominated behavior at 7.4 MPa

**Fig. 3:**
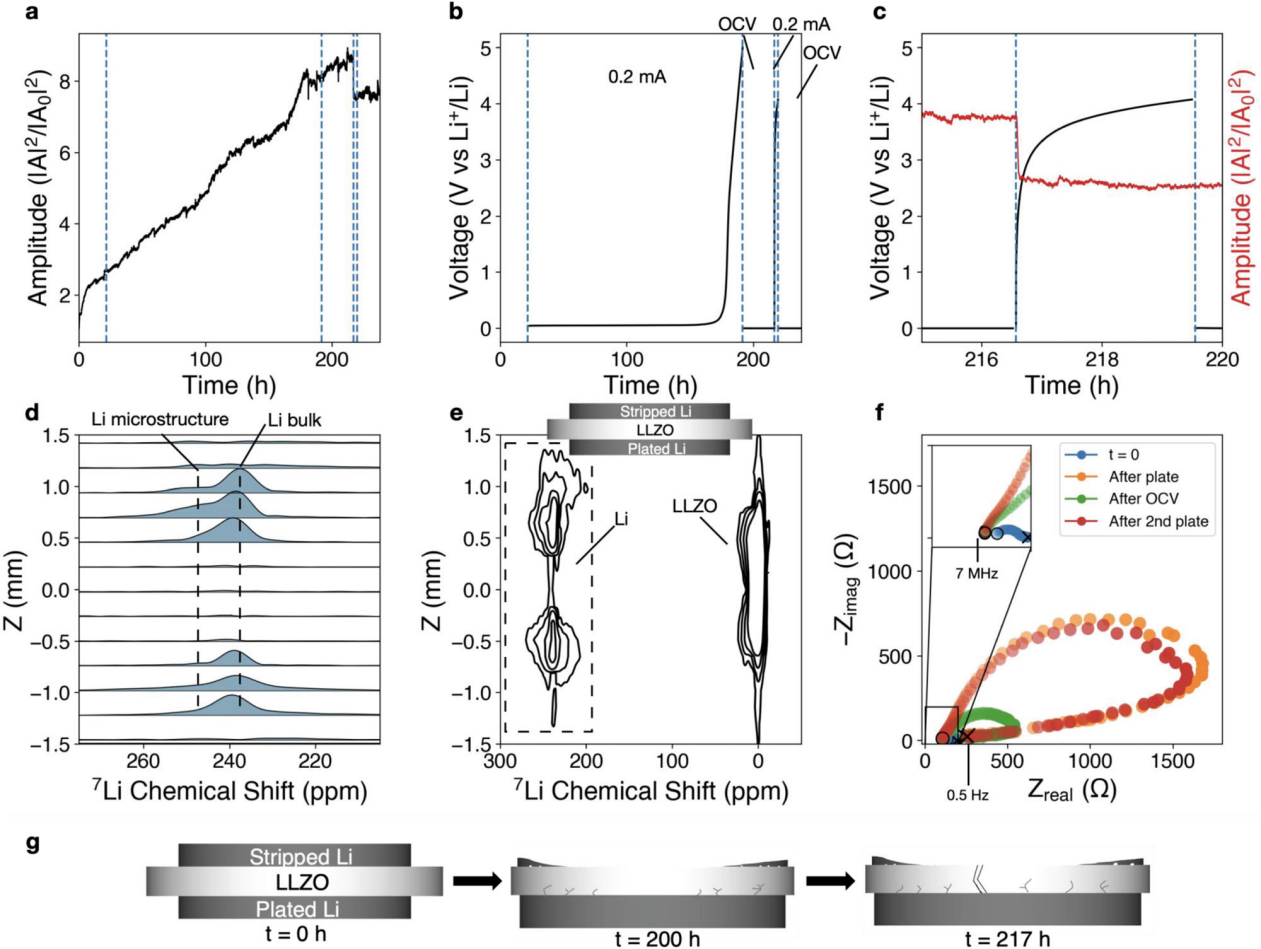
Lithium metal–LLZO interface at 7.4 MPa. Polarization test of Li (s)–LLZO interface at 7.4 MPa stack pressure conducted in a Li/LLZO/Li cell. After a few hours of initial mechanical equilibration, 0.2 mA/cm^2^ is applied galvanostatically until the voltage cutoff of 5 V, followed by a 25 h OCV rest step, a second polarization step in the same direction, and a second OCV rest step. **a** Normalized acoustic amplitude. **b** Voltage profile showing an increase in overpotential to voltage cutoff of 5 V during the galvanostatic current step of 0.2 mA/cm^2^. Vertical guidelines (dotted blue lines) are shown as a visual aid to delineate start and stop of galvanostatic current. **c** Zoomed in region of second polarization step which is accompanied by a sudden attenuation of acoustic amplitude. **d** 1D slices of ex situ ^7^Li CSI highlighting the lithium metal region between 200 and 280 ppm, as indicated by the dashed region in **e**. **e** Ex situ ^7^Li CSI contour plot showing microstructural lithium signal at the Li (s)–LLZO interface for both electrodes, as well as a small quantity of lithium metal signal extending through the body of the electrolyte. **f** Nyquist plot from potentiostatic electrochemical impedance spectroscopy conducted during initial OCV, after galvanostatic current to the voltage cutoff, after the 25 h OCV, and after the second plating step to the voltage cutoff. The open circle indicates the high-frequency start point (7 MHz), and the cross indicates the low-frequency end point (0.5 Hz). **g** Simplified cartoon illustration of interface behavior implied by the experimental data (not drawn to scale).

Upon increasing the stack pressure from 2 to 7.4 MPa, the acoustic amplitude no longer attenuates, but rather increases steadily over time (Fig. 3a). This higher pressure is well above the yield strength of 0.8 MPa for bulk lithium metal and causes the lithium foil to plastically deform (flatten) over time. The improved contact and decreased cell thickness results in a higher wave transmission amplitude. The enlarged contact area of the plastically deformed lithium foil is measured by EIS during the initial OCV before polarization, where decreasing cell impedance is observed (Supplementary Fig. 6a, b). The cell is able to galvanostatically plate most of the lithium foil from one side to the other until the polarization voltage cutoff of 5 V vs Li^+^/Li, after around 200 h of charge passed, equivalent to 40 mAh (~200 μm) (Fig. 3b). While amplitude continues to rise as lithium creep dominates mechanical change at this stack pressure, the 25 h OCV rest step does not result in complete recovery of cell impedance (see below). The initial cell impedance of ~250 Ω increases to over 1500 Ω after the first polarization step, recovers to around 500 Ω during the rest step, and then increases to over 1500 Ω after the second polarization step. While 7.4 MPa may be sufficient to prevent mesoscale-size interfacial contact loss during galvanostatic polarization, passing enough charge to the point of exposing LLZO to bare copper significantly alters lithium transport and local current densities.

At the onset of the second polarization step near *t* = 217 h (Fig. 3c), an instantaneous drop in amplitude was observed, though an internal short circuit was not detected electrochemically. The second cell polarization step began at a higher overpotential (>1 V vs Li^+^/Li) than the first, and the cell polarized to 5 V in less than 3 h. After cell disassembly, a large crack was visible to the naked eye through the center of the electrolyte, which most likely resulted in the sudden amplitude attenuation event. The crack may have been related to in-plane stress caused by heterogeneous volume change in the electrolyte due to nearly 40 mAh of charge passed in the first step. ^7^Li CSI reveals the presence of microstructural lithium on both electrodes (250–260 ppm), which is attributed to microscale voids that persist on the stripped lithium side and lithium protrusions near the plated lithium side. At first glance, the ^7^Li CSI contour plot appears to show a short circuit as indicated by the crossover of the lithium microstructure signal from the plated side to the stripped side (Fig. 3d, e). In other words, the signal at around 240 ppm connects the stripped side on top to the plated side on bottom. However, observation of the 1D slices from CSI in Fig. 3d indicates that the lithium microstructure peaks within the LLZO between 0.5 and −0.5 mm are minor; the lines shown in Fig. 3e are dependent on the contour levels. The presence of small growth asperities at local regions within the electrolyte is likely, due to the presence of a small amount of microstructure signal without an electrochemical short. In addition, it is possible that the lithium microstructures detected within the electrolyte nucleated at LLZO grain boundaries rather than the bulk electrode, as supported by EELS measurements of bandgap reduction at LLZO grain boundaries by Liu et al.^39^. ^7^Li CSI analysis at the LLZO–lithium interfaces shows a new resonance at ~0 ppm, consistent with LLZO decomposition products at the interphase. These interphasial products are correlated with   relatively constant impedance observed between the first and second plating (Fig. 3f), suggesting that these decomposition products stabilize the LLZO–lithium interface. Further discussion and confirmation of the interface chemistry can be found in Supplementary Discussion 1.3 and Supplementary Fig. 7a–f, with an illustration of overall behavior in Fig. 3g. Direct lithium deposition within LLZO was previously observed by Liu et al.^39^ and Han et al.^40^, and has been attributed to high local electronic conductivity and a reduced bandgap at LLZO grain boundaries. The reduced bandgap is also supported by DFT calculations indicating possible electrolyte oxidation at potentials as low as 3 V vs Li^+^/Li^4^.

### Creep-dominated behavior and crack propagation at 13 MPa

**Fig. 4:**
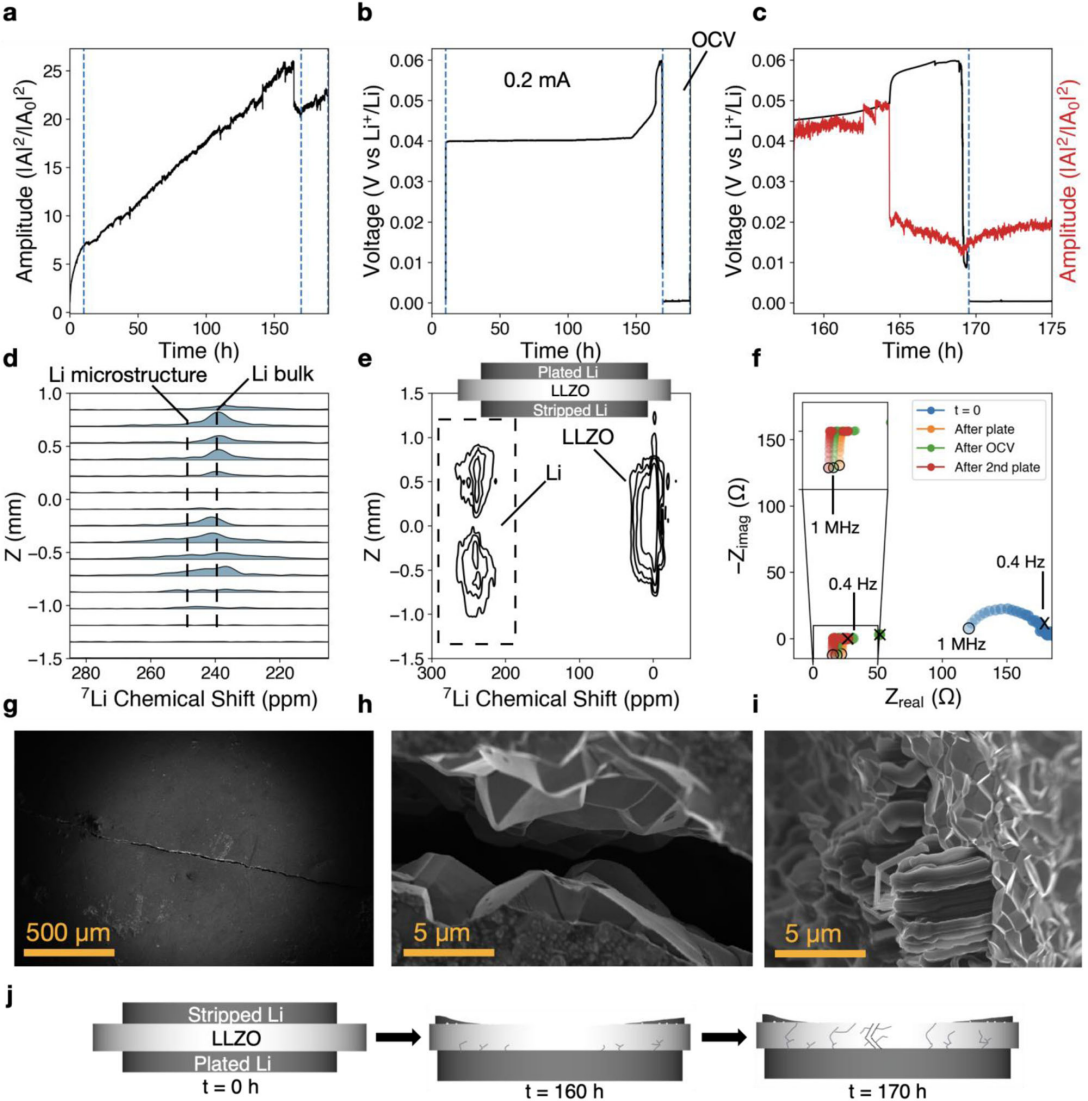
Lithium metal–LLZO interface at 13 MPa. Polarization test of Li (s)–LLZO interface at 13 MPa stack pressure conducted in a Li/LLZO/Li cell. After a few hours of initial mechanical equilibration, 0.2 mA/cm^2^ is applied galvanostatically until the voltage cutoff of 5 V, followed by a 25 h OCV rest step, a second polarization step in the same direction, and a second OCV rest step. **a** Normalized acoustic amplitude. **b** Voltage showing increase in overpotential until an electrochemical short at ~60 mV vs Li^+^/Li (*t* ~ 169 h) during the galvanostatic current step of 0.2 mA/cm^2^. Vertical guidelines (blue dotted lines) are shown as a visual aid to delineate start and stop of galvanostatic current. **c** Zoomed in region of electrochemical shorting event at around *t* = 169 h which is accompanied by a sudden attenuation of acoustic amplitude. **d** 1D slices of ex situ ^7^Li CSI highlighting the lithium metal peak between 200 and 280 ppm, as indicated by the dashed region in **e**. **e** Ex situ ^7^Li CSI contour plot showing formation of lithium microstructures at the Li (s)–LLZO interface for both electrodes, and the presence of bulk lithium metal (240 ppm) in the electrolyte. **f** Nyquist plot from potentiostatic electrochemical impedance spectroscopy conducted during initial OCV, after galvanostatic current to the voltage cutoff, after the 25 h OCV, and after the second plating step to the voltage cutoff. The open circle indicates the high-frequency start point (1 MHz), and the cross indicates the low-frequency end point (0.4 Hz). **g** SEM image of center of LLZO on the stripped lithium side (in this case, most of the existing lithium foil was plated through the electrolyte resulting in an almost-bare LLZO surface), showing a large crack. **h** High-resolution SEM image of crack which reveals the polycrystalline grains of LLZO. **i** High-resolution SEM image showing the presence of lithium protrusions from polycrystalline grain boundaries along the exposed crack, with protrusions oriented parallel to the electrode (instead of protruding through the electrolyte). **j** Simplified cartoon illustration of interface behavior implied by the experimental data; not drawn to scale.

A polarization test was conducted at an even higher stack pressure of 13 MPa (Fig. 4), which is near the high stack pressure regime determined by Zhang and Harris’ contact model for maintaining a conformal interface. At 13 MPa, the acoustic amplitude continues to increase during galvanostatic charging (Fig. 4a, b). The high stack pressure causes the entire lithium foil to yield and plastically deform (flatten) against the LLZO. The plated lithium foil side experiences an increase in area with approximately twice the diameter after over 150 h of plating. Cell disassembly indicated that nearly the entire foil had plated through the electrolyte and the LLZO surface on the stripped side was visible. The acoustic amplitude progressively increases in intensity during the long plating step. At around *t* = 164 h, a sudden attenuation in amplitude is observed, which correlates with an 8 mV jump in overpotential (Fig. 4c). Five hours later, the voltage falls from 60 to less than 10 mV, indicating an electrochemical short. This agrees with post-mortem ^7^Li CSI experiments that show lithium propagation through the electrolyte (Fig. 4d, e), as well as EIS measurements that show a ten-fold impedance drop from 200 to 20 Ω after polarization (Fig. 4f). The CSI contour plot (Fig. 4e) indicates that microstructural ^7^Li signal at ~253 ppm is observed in conjunction with the bulk lithium metal signal at ~240 ppm at various *z*-positions at both electrodes (the top electrode is the plated lithium, the bottom is the stripped side). Within the electrolyte, the presence of bulk lithium metal signal (240 ppm) instead of microstructural lithium signal (250–260 ppm) suggests that lithium microstructures adopt a planar morphology within the electrolyte. SEM imaging within the large crack through the middle of the LLZO (Fig. 4g) revealed the presence of lithium growth protruding from the sides. These growths are oriented parallel to the electrode and are visibly differentiated from the polycrystalline LLZO (Fig. 4h, i). Supplementary Figure 8 explains the locations on the cell where the SEM images were taken; the crack occurs through the center of the cell, and images are taken within the crack where the polycrystalline LLZO along the sides are visible.

Taken together, these results suggest stable plating and stripping until around *t* = 150 h when the overpotential begins to increase (Fig. 4j). The large structural crack likely initiated at *t* = 164 h, given the sudden attenuation in acoustic amplitude and the simultaneous jump in overpotential. This behavior is followed by the electrochemical short at *t* = 169 h. Lithium growth near the surface of the plated side and microscale voids on the stripped side likely formed before the crack event due to the abundance of microstructure on both electrodes observed in ^7^Li CSI (Fig. 4e). The presence of bulk lithium metal signal (~240 ppm) within the electrolyte contrasts with the typically observed microstructural lithium metal signal (~250–260 ppm) in the electrolyte due to filament propagation perpendicular to the electrode, but agrees with the post-mortem SEM images indicating propagation parallel to the electrode (Fig. 4i and Supplementary Fig. 8). The uneven displacement of lithium throughout 170 h of charge passed, the growth of lithium through the electrolyte, and the current collector directly in contact with LLZO would have all contributed to current focusing effects, leading to the crack, lateral lithium growth and extrusion within the electrolyte, and the eventual electrochemical short at 169 h. We hypothesize that higher stack pressures increase stress in the lateral direction, or parallel to the Li (s)–LLZO interface, which may influence the preferred orientation of lithium propagation. These results further support the notion that while a high stack pressure of 13 MPa (or even 7 MPa) improves bulk contact at the interface, it can also lead to other degradation modes, such as the observed laterally oriented lithium penetration into the electrolyte and subsequent electrolyte fracture. As shown in Supplementary Fig. 9, shifts to higher frequency (~11 ppm) of the LLZO peak in the ^7^Li CSI indicate electron-nuclear coupling arising from a lithium-ion intercalated interphase. We observe a similarly conductive interphase when polarizing at low pressure (2 MPa, Fig. 2 and Supplementary Fig. 3), yet the interphase formed at medium pressure (7.4 MPa, Fig. 3 and Supplementary Fig. 7) is comprised of LLZO decomposition products that appear to be electronically insulating. We suspect that the cell at 7.4 MPa generated more LLZO decomposition products due to increased time spent at higher voltages (~200 h required for polarization to 5 V, without shorting). In contrast, the cell at 2 MPa polarized faster (~5 h) and did not allow sufficient time for measurable LLZO decomposition to occur. Likewise, the cell at 13 MPa short-circuited at 0.06 V, preventing LLZO decomposition at high overpotentials. Kazyak et al.^14^ also visualized lateral growth of lithium in an optical cell, but the pressure, geometry, and polarization is significantly different, and it is unclear whether similar mechanisms are at play. Our results agree with contact modeling predictions by Tu et al.^41^, which suggest large stack pressures may lead to lithium infiltration into grain boundaries and possibly even solid electrolyte fracture, though a higher stack pressure of 160 MPa was predicted to exceed the solid electrolyte fracture toughness. The observed electrolyte fracture at comparably lower stack pressures in our experimental results may be affected by the non-uniform stripping of lithium down to copper that is not considered in the model. Thus, the ability of LLZO to prevent lithium penetration without further cell engineering is unclear, as metallic lithium crossover can occur in a variety of conditions.

### Void formation and creep behavior at 0.5 mA/cm^2^

**Fig. 5:**
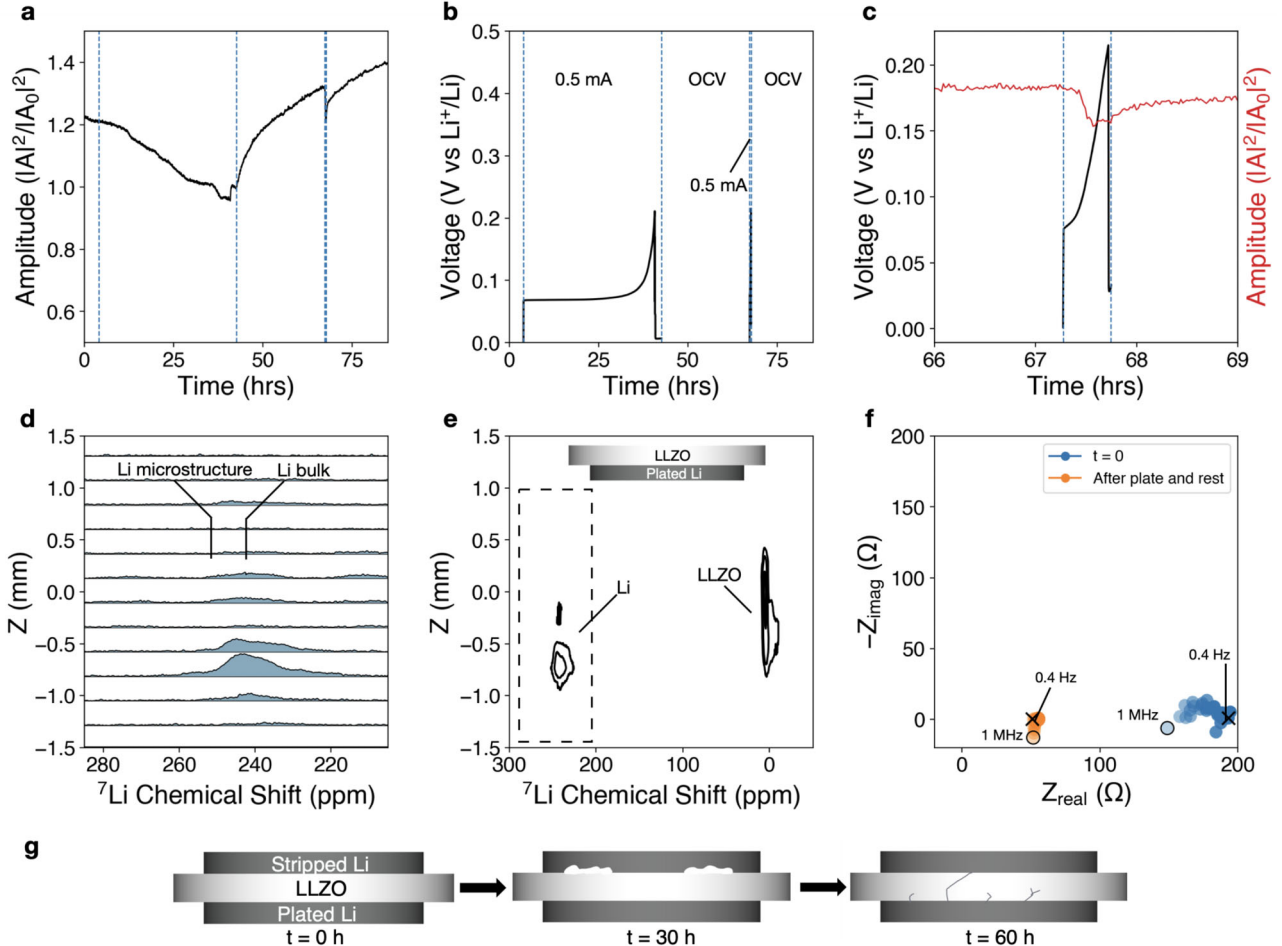
Lithium metal–LLZO interface at 0.5 mA/cm^2^. Polarization test of Li (s)–LLZO interface at 7.4 MPa stack pressure conducted in a Li/LLZO/Li cell. After a few hours of initial mechanical equilibration, 0.5 mA/cm^2^ is applied galvanostatically, followed by a 25 h OCV rest step, a second polarization step in the same direction, and a second OCV rest step. **a** Normalized acoustic amplitude. **b** Voltage curve depicting two polarization steps and cell shorting at 0.2 V vs Li^+^/Li. Vertical guidelines are shown as a visual aid to delineate start and stop of galvanostatic current. **c** Zoomed in region of second polarization step showing a starting overpotential of ~75 mV but rapid polarization and another electrochemical short. **d** 1D slices of ex situ ^7^Li CSI highlighting lithium metal signal between 200 and 280 ppm, as indicated by the dashed region in (**e**). **e** Ex situ ^7^Li CSI contour plot showing small amounts of lithium penetration into the electrolyte, with the stripped lithium side (top) having fallen off after cell disassembly. **f** Nyquist plot from potentiostatic electrochemical impedance spectroscopy conducted during initial OCV, and after the electrochemical short. The open circle indicates the high-frequency start point (1 MHz), and the cross indicates the low-frequency end point (0.4 Hz). **g** Simplified cartoon illustration of interface behavior implied by the experimental data; not drawn to scale.

Given the lack of amplitude attenuation at 7.4 and 13 MPa when compared with 2 MPa, polarization tests were conducted at a higher current density of 0.5 mA/cm^2^. At 7.4 MPa, the acoustic amplitude attenuated shortly after applying galvanostatic current, as observed in Fig. 5a. In comparison with the low current test at 7.4 MPa (Fig. 3) or the CCD test at 7.4 MPa (Supplementary Fig. 2), this higher current density resulted in a ~20% loss of amplitude rather than a continued increase as observed for creep-dominated mechanics at 0.2 mA/cm^2^. The amplitude immediately recovered during the subsequent OCV step (*t* ~ 40 h in Fig. 5a), with an overall increase attributed to the thinning of lithium foil at this stack pressure. The second polarization step resulted in a starting overpotential near the initial overpotential of the cell, with a difference of ~10 mV (Fig. 5b, c). However, in both polarization steps, the cell presented a short at ~0.2 V before reaching the voltage cutoff. The amplitude stopped attenuating after the electrochemical short, since void formation also ceases without the applied overpotential. ^7^Li CSI (Fig. 5d, e) demonstrated less microstructure content in comparison with the 0.2 mA/cm^2^ tests. The predominant failure mode appears to be sudden and localized electrochemical shorting as observed by the voltage drop and lower cell impedance (200 to 50 Ω) at the end of the test (Fig. 5f), rather than full polarization to the voltage cutoff with significant microstructure growth over a longer duration of charge passed, as for the lower current tests. Despite the lack of significant microstructure growth or macroscopic interfacial contact loss, this cell still polarized rapidly during the second current step, which could be attributed to electrolyte degradation from local shorting (Fig. 5g). Minimal chemical change at the interphase was observed in the ^7^Li CSI electrolyte peak (Supplementary Fig. 10a–f), likely due to the brevity of the test and the short circuit which prevented polarization to voltages higher than 0.2 V vs Li^+^/Li.

### Degradation mechanisms at various stack pressure regimes

The usage of acoustic transmission to detect loss of interfacial contact and subsequent ^7^Li CSI to spatially resolve the degree of microstructure formation provides insights into the dynamics of the Li (s)–LLZO interface over long durations of charge and throughout a wide range of stack pressure regimes. A relatively low stack pressure of 2 MPa resulted in amplitude attenuation to ~80% of the initial intensity and minimal recovery back to ~82% of the initial intensity during a 25 h rest step, along with significant microstructure buildup. Higher stack pressures of 7.4 and 13 MPa resulted in a mechanically stable interface as measured by the lack of acoustic amplitude attenuation, but still caused microstructure formation and a sudden amplitude attenuation event after passing a significant amount of charge. The enhanced interfacial stability at higher stack pressures results in over 40 mAh of charge passed until cell polarization. On the other hand, a higher current density of 0.5 mA/cm^2^ may increase the propensity for electrochemical shorting due to local lithium penetration. Lastly, these far-from-equilibrium operating conditions such as high voltage or high current densities may also lead to chemical degradation forming interphase compounds, as corroborated by ^7^Li CSI analysis of the electrolyte signal and EDS. DFT calculations by Thompson et al.^42^ suggest an electrochemical stability window of up to 5 V vs Li^+^/Li. In contrast, other studies have suggested a lower chemical stability window, including electrolyte oxidation to Li_2_O_2_, La_2_O_3_, Li_6_Zr_2_O_7_ at potentials as low as 3 V vs Li^+^/Li^4^. Probing these subtle chemical changes requires high resolution and localized chemical characterization, such as a recent study by Liu et al.^39^ using HRTEM and EELS that suggested bandgap reduction at LLZO grain boundaries and subtle changes in oxygen concentration. In the present study, the observable but minimal amount of chemical changes in CSI and EDS, along with the wide range of applied stack pressure, suggests that dominant changes are mechanical with some degree of chemical effects. Besides the minor deposition and growth of lithium within LLZO, the vast majority of the deposited lithium occurs at the electrode bulk, as observed by a roughened interface at the plated lithium side in cross-sectional SEM images (Supplementary Figs. 3 and  10).

### Synchronized operando acoustic transmission and ssNMR spectroscopy

**Fig. 6:**
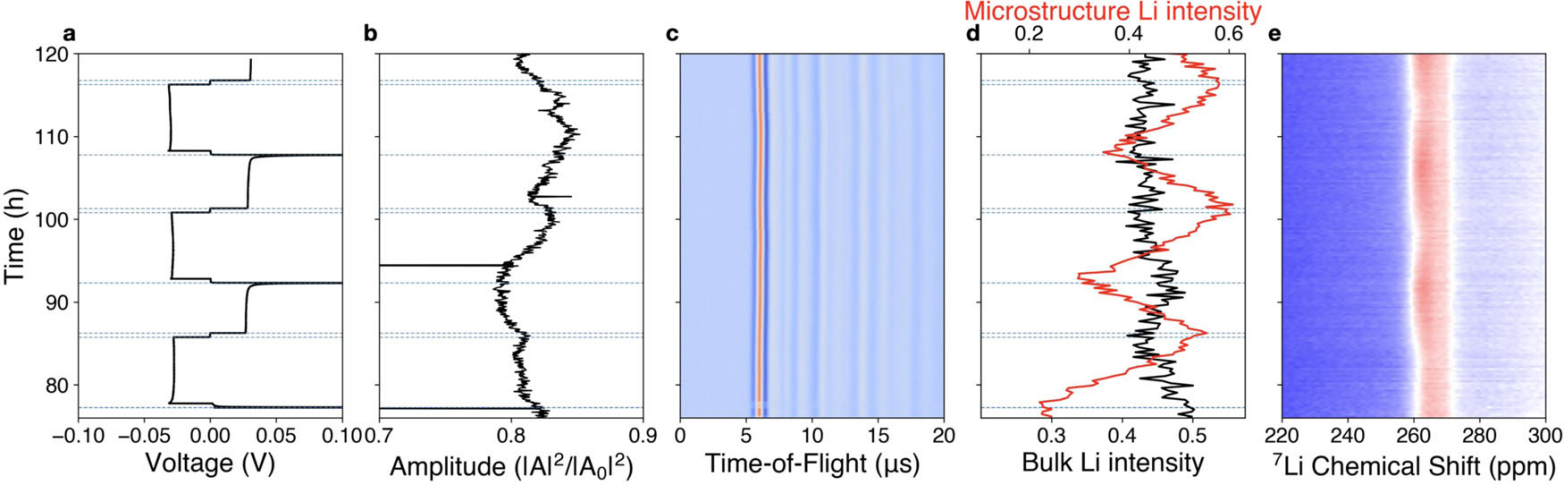
Synchronized operando acoustic transmission and ssNMR spectroscopy. Lithium plating and stripping through LLZO at a current rate of 0.05 mA/cm^2^ in a Li/LLZO/Cu cell^43^, indicating microstructure formation during plating on copper and irreversible microstructure formation over three cycles. ssNMR and acoustic transmission are conducted simultaneously, as shown by the schematic in Fig. 1b. **a** Voltage (V vs Li^+^/Li) profile, **b** acoustic transmission amplitude (arbitrary units), **c** acoustic time-of-flight (μs) of the entire waveform, **d** ssNMR normalized peak intensities, **e** ^7^Li chemical shift (ppm) of the lithium metal region.

Following the constant stack pressure polarization tests, we designed a synchronized operando acoustic/ssNMR cell and conducted measurements to correlate the dynamic changes in interfacial contact with the real-time rate of microstructure formation (Fig. 6). For this setup, both cell mechanics and microstructure formation are measured at the same time, for a Li/LLZO/Cu cell configuration. Specifically, the cell was placed inside a cell holder in the NMR probe, and both acoustic transducers and electrochemical leads were attached. Acoustic pulses were applied continuously, collecting waveforms every second, while NMR radio frequency (r.f.) pulses were applied separately, taking approximately 17 min for each full 1D scan. The combined electrochemical (Fig. 6a), acoustic (Fig. 6b, c), and NMR (Fig. 6d, e) data were time synchronized during data analysis. Detailed procedures are described in the “Methods” section.

Lithium deposition and stripping from a Cu–LLZO interface results in increased microstructural growth as compared with a Li (s)–LLZO interface; conformal attachment of the copper foil to the LLZO surface is previously described by Wang et al.^43^. In order to provide enough temporal resolution for operando measurements, 1D ssNMR was used rather than ^7^Li CSI. During the initial plating step of a Li/LLZO/Cu cell housed within the NMR magnet in a fixed gap cell, we observe increased lithium microstructural content for the ^7^Li resonance centered at ~258 ppm (Fig. 6d, e). This increase in the microstructural peak intensity is attributed to the non-planarity of plated lithium on the bare copper current collector. The intensity of the lithium microstructure peak then decreases upon the subsequent stripping current but does not completely disappear. The persistence of lithium microstructural signal (Fig. 6d, red curve indicates lithium microstructural peak intensity) after stripping to the voltage cutoff arises from incomplete and non-uniform stripping of lithium from the copper surface. In contrast to the fixed pressure cell, the stack pressure of the synchronized operando cell inside the NMR magnet arises from hand-tightened threads on the cell body and is likely in the sub-1 MPa range (Fig. 1b). We estimate the stack pressure of the operando cell from EIS measurements, where the operando cell shows an impedance between 240 and 270 Ω, as opposed to 220 Ω at 2 MPa and 170 Ω at 7.4 MPa in the fixed pressure cell. A fixed gap cell geometry is more comparable to typical commercial cell conditions, and multiple plating and stripping steps likely lead to increased cell pressure over cycling. The complex balance between steadily increasing pressure and roughening of the lithium–LLZO interfaces manifests as a relatively steady acoustic amplitude (Fig. 6a–c).

We demonstrate a proof-of-concept synchronized operando setup that can probe both the microstructure and cell mechanics of a buried interface during cycling. This is useful for investigating dynamic cell behavior free from cell-to-cell variations for all types of cell chemistries and materials held at relatively low stack pressure.

The results herein present a multimodal and dynamic mechanistic analysis of the Li (s)–LLZO interface for solid-state batteries. Changes in both interfacial contact and microstructural growth are characterized in operando using complementary acoustic transmission and ssNMR/CSI. The results indicate that a stack pressure of 2 MPa is insufficient for maintaining a conformal Li (s)–LLZO interface at 0.2 mA/cm^2^ or higher due to continuous void formation on the stripped lithium side, resulting in almost immediate acoustic amplitude attenuation and significant surface roughening. A stack pressure of 7 MPa and higher leads to creep-driven interfacial dynamics but does not indefinitely eliminate microstructure growth. ^7^Li CSI shows that the lithium microstructure formed within the electrolyte is oriented laterally to the electrodes. A high stack pressure of 13 MPa increases the likelihood of exceeding the local yield stress to the point of fracture and cell shorting. Sudden mechanical changes most likely due to crack propagation are detectable with operando acoustic transmission. In all cases, both electrochemical and acoustic signals show hysteresis despite a long OCV hold, indicating the lack of complete interfacial stability. The large swings in impedance ranging from short circuit to >6000 Ω indicate that the primary failure modes are mechanically driven due to the wide range of operating stack pressures, but local chemical heterogeneities may also be influential to some degree when driven to high overpotentials, and may be further studied with spatially resolved chemical analyses. A proof-of-concept synchronized operando acoustic/ssNMR characterization approach is used to measure both interfacial and microstructural changes at the same time. The non-invasive, non-destructive, and complementary usage of both acoustic transmission and ssNMR can be generalized for dynamically characterizing both mechanics and microstructural heterogeneity of other buried electrode and solid-state electrolyte interfaces. The operando chemo-mechanical data gathered at various stack pressure regimes can be incorporated into existing contact models, and further point toward the need for low stack pressure operation given the intrinsic degradation modes that are observed to arise at high stack pressures and long durations of charge passed.

## Methods

### Cell assembly and electrochemistry

LLZO pellets were synthesized by rapid induction hot-pressing with details referenced in previous literature^7,32^. Pellets were cut on a diamond saw to a thickness of ~1.3 mm for Li/LLZO/Li cells and ~2.0 mm for Li/LLZO/Cu cells. The LLZO pellets were polished with sandpaper (from 600 grit and progressively finer to 1200 grit) and a ~0.1 μm diamond paste, stored in an Argon glovebox (H_2_O < 0.5 ppm, O_2_ < 0.5 ppm) and heat treated at 400 °C for 3 h to remove Li_2_CO_3_ surface contamination. For Li/LLZO/Li cells, lithium metal foil (500 μm thickness, 99.9% purity, Alfa-Aesar) was pressed on both sides of the polished pellet surface under a load of 3.2 MPa and then heated at 170 °C under a load of ~1 MPa for optimal interfacial contact. Pressure was applied with a custom-built cell fixture using a spring gauge to control the applied load and an insulated hot plate to apply heat. For Li/LLZO/Cu cells, the metal current collector (10-μm-thick battery-grade copper, Targray) was laminated to the polished LLZO surface by applying a pressure of 0.8 MPa at 900 °C for 5 min^43^. Battery-grade copper (10 μm) foils were purchased from Targray and used as current collectors. EIS and voltage polarization tests were conducted with a potentiostat (Gamry Instruments 3000). EIS was conducted in potentiostatic mode from 7 MHz to 500 mHz with 120 mV amplitude and 14 points per decade. The fixed pressure cell tests were conducted in a temperature chamber (Neware) at 30 °C.

### Scanning electron microscopy

SEM imaging was conducted after cell disassembly (Zeiss Sigma VP SEM). The tabs were removed, and the cell was placed on a sample stub within an Argon-filled glovebox. The stub was transferred to the SEM in transfer vessels to prevent exposure to ambient atmosphere. Most cycling conditions resulted in an exposed LLZO surface on the stripped side, either due to delamination at low stack pressures or complete stripping at high stack pressures. This exposed LLZO surface was then imaged at 12 kV. EDS was conducted with the attached Bruker EDS at 12 kV. SEM/EDS cross-sectional images and chemical analyses were conducted by cross-sectioning the cell with a stainless steel blade and mounting on a 90° angle on the stainless steel SEM stub. For example, Supplementary Fig. 11 depicts a clean cross-section of a cell that had been polarized to 5 V at 0.5 mA/cm^2^, leading to delamination of the stripping side but an intact interface at the plating interface.

### Operando acoustic transmission

Two wide-band ultrasound transducers (Olympus) with central frequency of 2.25 MHz and 10 mm beam diameter were fixed in place within a double-piston pneumatic cylinder and spacers made of cross-linked polystyrene (Rexolite, a low acoustic attenuation material). The pneumatic cylinder is actuated with a solenoid controlled with an Arduino to apply constant pressure, and the spacers act as both force distribution plates as well as a fixed distance for acoustic transmission beyond the near-field. Acoustic signals were produced with an ultrasonic pulser (US Ultratek, Inc.) in transmission mode with 300 V impulse spikes of pulse width corresponding to the central frequency of the transducer, and measured with a high-resolution oscilloscope sampling at 1 GS/s (Picoscope). The physical hardware was controlled with Linux-based isolated and virtualized software operating systems (Docker) to ensure reliable and accurate fast data collection. Detailed explanation of the setup and technique is described elsewhere by Chang et al. and open source code can be found at https://github.com/dansteingart/.^21,22^

### Nuclear magnetic resonance spectroscopy and ^7^Li CSI

All ^7^Li ssNMR and CSI measurements were performed on a Bruker 400 Avance NEO spectrometer operating at 9.4 T (^7^Li frequency = 155.45 MHz) equipped with a 25 mm ^1^H/^7^Li Bruker MICWB40 probehead and a triple axis micro2.5 gradient system. ^7^Li chemical shifts were internally referenced to LLZO at 2.3 ppm. Prior to analysis, cells were sealed in an airtight vial under argon and oriented with the lithium metal electrodes perpendicular to the external magnetic field, *B*_*0*_.

^7^Li CSI measurements were performed using a single phase-encoded spatial dimension, along *z* (*B*_*0*_), as reported previously^44^. After a 90° r.f.   pulse applied on the ^7^Li channel (34.6 μs at 200 W), a phase-encoding gradient was applied along the *z*-axis followed by a 180° r.f. pulse with a recycle delay of 0.5 s. One-dimensional ^7^Li ssNMR measurements were performed using a 90° pulse length of 78.9 μs at 30 W.

All NMR data were acquired and processed using Bruker Topspin 4.1.1 software. Data were plotted using the Python 3 package *matplotlib*, with a separate package *nmrglue* used to interface between Topspin and Python.

### Synchronized operando acoustic and ssNMR characterization

Two wide-band ultrasonic transducers (Olympus) with central frequency of 2.25 MHz and 10 mm beam diameter were firmly epoxied on either side of a machined polytetrafluroethylene (PTFE) cell holder, with acoustic gel couplant (Sonogel) placed between the transducer face and the PTFE cell surface (Fig. 1b). The fabricated cells were placed inside the cell holder inside an argon-filled glovebox (O_2_ < 0.5 ppm, H_2_O < 0.5 ppm) with thin strips of battery-grade copper mesh current collector (99.5%, Goodfellow) instead of nickel foil to minimize the amount of metal used. The ultrasonic transducers were attached to double-shielded type RG314 cables, and shielded electrochemical cables were attached onto the protruding copper mesh current collectors by using a minimal amount of tin solder between the exposed wiring and the edge of the copper mesh. The cell potential was checked with a multimeter to ensure good contact was achieved, before the operando tests. Typically, loss of electrochemical contact was due to tightening the cell threads and tearing the copper mesh foil inside the cell holder. Both the ultrasonic and the electrochemical cables were connected outside the magnet to a gold shielding plate with 15 MHz low-pass filters before connection to the corresponding instruments as a precaution, though we did not observe significant changes to the signal-to-noise with or without the filters. The primary change in reducing the signal-to-noise was threading the cabling on the bottom electrode down through the magnet, and the cabling connected to the top electrode up through the top of the magnet, such that no cabling crossed the electrolyte area.

While typical ex situ ^7^Li experiments are performed using a r.f. pulse power of 200 W to maximize sensitivity, we found that strong r.f. fields (>50 W) interfered with acoustic signal collection, causing intermittent noise or entirely disconnecting the acoustic pulser^45^. Resetting the r.f. pulse sequence with the transducers inside the magnet also appeared to interfere with acoustic data collection, and care was taken to start acoustic pulsing before moving the cell holder into the magnet. A custom script was used to continuously pulse acoustic waveforms at regular time intervals (e.g., once every second), and then read waveforms from the oscilloscope; starting the continuous pulse sequence while outside the magnet allowed for stable and uninterrupted acoustic data collection. Reducing the r.f. pulse power to 30 W also allowed for stable and uninterrupted acoustic data collection and NMR sensitivity sufficiently high for quantitative analysis. To clarify the order of each step, here is an example: the acoustic pulse sequence was started at *t* = 0 h with the cell inside the magnet probe and began to collect one averaged waveform every second, the probe was placed inside the NMR magnet at *t* = 0.1 h, the NMR pulse sequence was started at *t* = 0.5 h and began to collect one signal-averaged 1D NMR experiment every 17 min, and finally the electrochemistry was started at *t* = 5 h after some initial acoustic signal equilibration.

## Supplementary information

Supplementary Information

## Data Availability

The datasets generated during this study are available from the corresponding authors (lem2221@columbia.edu, dan.steingart@columbia.edu) on reasonable request.

## Code availability

The code used for data analysis in this study is available from the corresponding authors on reasonable request.
