# Supplementary material for: Evolving contact mechanics and microstructure formation dynamics of the lithium metal-Li7La3Zr2O12 interface
Source: Nat Commun. 2021 Nov 4;12:6369. doi: 10.1038/s41467-021-26632-x (PMC8569160; doi:10.1038/s41467-021-26632-x)
Supplement: Supplementary file 1 — Supplementary Information [file 41467_2021_26632_MOESM1_ESM.pdf]

SUPPLEMENTARY INFORMATION

Evolving Contact Mechanics and Microstructure Formation Dynamics of the Lithium Metal-$Li_7La_3Zr_2O_{12}$ Interface

Wesley Chang[1,2,3,4], Richard May[3,4], Michael Wang[5], Gunnar Thorsteinsson[6], Jeff Sakamoto[5,7], Lauren Marbella[3,4,*], Daniel Steingart[3,4,6,*]

[1]Department of Mechanical and Aerospace Engineering, Princeton University, Princeton, NJ 08544

[2]Andlinger Center for Energy and the Environment, Princeton University, Princeton, NJ 08544

[3]Department of Chemical Engineering, Columbia University, New York, NY 10027

[4]Columbia Electrochemical Energy Center, Columbia University, New York, NY 10027

[5]Department of Materials Science and Engineering, University of Michigan, Ann Arbor, MI 48104

[6]Department of Earth and Environmental Engineering, Columbia University, New York, NY 10027

[7]Department of Mechanical Engineering, University of Michigan, Ann Arbor, MI 48104

[*]Corresponding Authors
lem2221@columbia.edu, dan.steingart@columbia.edu

These authors contributed equally: Wesley Chang, Richard May

These authors jointly supervised this work: Lauren Marbella, Daniel Steingart

Supplementary Discussion

1.1 Explanation of acoustic effects of batteries.

Acoustic ultrasound is the propagation of high frequency sound waves, which are mechanical vibrations occurring within a fluid or solid. A recent comprehensive review paper by Majasan et al. is particularly useful for a general overview of acoustics for battery characterization.[1] Prior to 2015, most studies involved the passive observation of acoustic emission through electrochemical cells.[2] Gold et al. and Hsieh et al. presented work on the use of acoustic transmission to actively detect changes in the physical state of a cell.[3,4] In transmission mode, a sound wave of ultrasound frequencies (typically 1 to 10 MHz) is generated from a piezoelectric transducer and propagated through the medium towards a receiving transducer. The sound wave contains a central frequency, an amplitude, and travels at a certain speed (in metal foils, usually > 4000 m/s; in a cell stack,

between 1000 to 2000 m/s). Our recent work by Chang et al. is a useful resource for understanding how the acoustic waveform signals are processed in order to obtain quantitative information such as material stiffness and sound speed.[5] In the context of this work, the total amplitude as calculated and plotted in each of the main figures is the integral of the waveform, therefore capturing any change in the waveform intensity. The time-of-flight (ToF) is the time it takes for the sound wave to propagate through the medium, and is related to the thickness and sound speed by the following equation:

$ToF = \frac{L}{C_s} = \frac{L}{\sqrt{E/\rho}}$ where L is thickness, E is modulus, and ρ is density

For a cell stack which contains materials each with different bulk moduli and density, an averaged approach is generally taken (e.g., in geophysics, a Backus average takes the harmonic average of each material to estimate the overall property of the heterogeneously layered structure).[5,6] In fluids, the modulus is the bulk modulus, which is the volume change of the fluid under an applied hydrostatic pressure. In solids, waves propagate both longitudinally (primary, compressional wave) and transversely (secondary, shear wave), and therefore, the modulus is a function of both the bulk modulus and shear modulus. Generally, shear waves arrive much later than longitudinal waves, and the primary wave that is measured is the longitudinal wave. When the wave strikes an interface, it will typically be reflected and transmitted simultaneously. The degree of reflection vs transmission is related to the acoustic impedance of the material, and this effect along every interface ultimately results in the measured transmitted wave amplitude.

For operando acoustic studies, waveforms are continuously pulsed through the medium, with each waveform recorded on an oscilloscope. Individual waveforms can be superimposed on a heatmap to show the evolution over time. Likewise, total amplitudes of each waveform can be plotted to determine the change in total amplitude as a function of cell cycling (such as the current study).

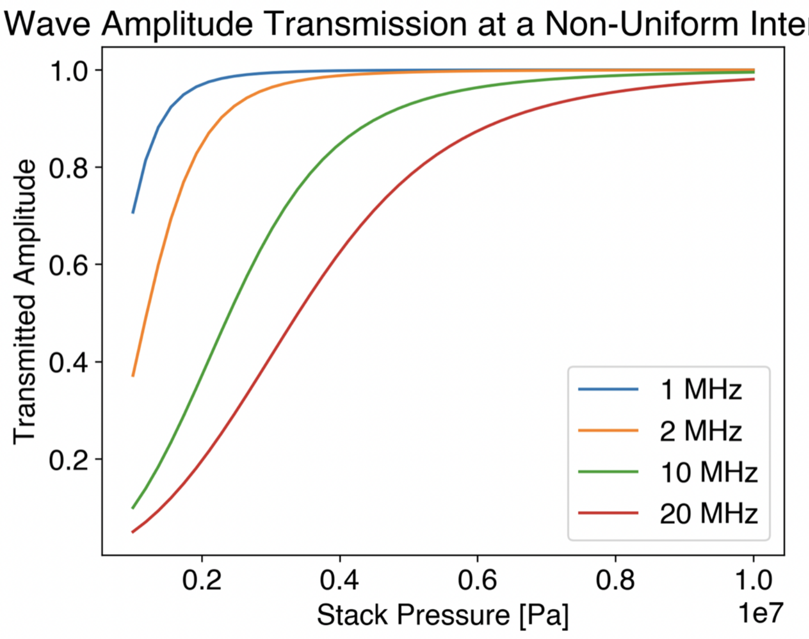

Supplementary Figure 1. Plot of transmitted wave amplitude vs stack pressure, from the finite-difference solution of the 1D acoustic wave equation with a dispersion term to account for interfacial contact loss (adapted from reference[7] for solid-solid interfaces). This model shows that a decrease in stack pressure at any wave frequency results in a loss of transmitted amplitude due to formation of a rough interface (which impacts the dispersion term).

In the current study, there are no bulk phase changes in the electrodes or the electrolyte (as opposed to graphite intercalation or lithium metal oxide cathode phase changes in lithium-ion batteries). Therefore, the primary contributor to acoustic amplitude attenuation is interfacial roughening due void formation at the stripping interface. As shown in the SEM images (e.g. Supplementary Figure 3), the plated interface, while increasingly non-uniform, is fully intact after the polarization tests. The voids on the stripping electrode side will cause the wave to attenuate due to a lower transmission efficiency of a solid/gas interface, and therefore the degree of interfacial contact loss can be correlated with amplitude attenuation. While a full model to quantify this effect is out of scope of the current study, we show a relation between stack pressure and amplitude attenuation modeled using a finite difference scheme to solve the 1D acoustic wave equation with a dispersion term (see Supplementary Figure 1). This serves to demonstrate the potential quantitative nature of acoustic characterization for detecting the degree of contact loss at buried interfaces. The model shows consistency with the data in the study, which utilizes a 2.5 MHz central frequency. An increase in stack pressure improves interfacial contact and increases the predicted transmitted amplitude. For example, a 2 MHz wave at a stack pressure of 2 MPa will transmit amplitude at roughly 80% of that at 5 MHz.

1.2 Explanation of bulk magnetic susceptibility (BMS) effects.

Lithium metal exhibits a unique chemical shift depending on its orientation in the external magnetic field. This phenomenon arises due to bulk magnetic susceptibility (BMS) effects from Pauli paramagnetism in lithium metal. For example, a strip of lithium metal oriented perpendicular to the external magnetic field gives a ^{7}Li shift of 242 ppm. In contrast, if the lithium strip is oriented parallel to the field, the shift for lithium metal is at 272 ppm. This orientation-dependent ^{7}Li shift for lithium metal provides a sensitive readout of sample orientation during the experiment, as well as a convenient way to probe lithium microstructural growth, which is oriented perpendicular to the lithium metal electrode.

1.3 Discussion of chemical analysis at the lithium metal - LLZO interface.

^{7}Li NMR spectra at the lithium - LLZO interface in CSI experiments provide information regarding the chemical species at that interface. Shifts to lower frequency as compared to LLZO (which appears at 2.3 ppm), such as those shown in Supplementary Figure 7 and Supplementary Figure 10, likely correspond to LLZO decomposition products. Conversely, shifts to higher frequency indicate electron-nuclear coupling which arises from formation of conductive species that likely indicate a Li^{+} intercalated interphase, such as those shown in Supplementary Figure 3 and Supplementary Figure 9. We see that lower stack pressures (2 MPa, Supplementary Figure 3) and higher stack pressures (13 MPa, Supplementary Figure 9) result in a more conductive Li^{+}-intercalated interphase, while a medium stack pressure (7.4 MPa, Supplementary Figure 7 and Supplementary Figure 10) results in LLZO decomposition products. Energy dispersive X-ray spectroscopy (EDS) of the cross-section further confirms possible surface reactions, exhibiting higher concentrations of oxygen at the plated lithium metal – LLZO interface in the elemental mapping (Supplementary Figure 3d). SEM of the cross-section indicates that the plated lithium – LLZO interface remains fully contacted after cross-sectioning, with some interfacial roughness due to uneven plating and which agrees with the microstructural signal observed in the ^{7}Li CSI (Supplementary Figure 3b). Relative concentrations of lanthanum, zirconium and oxygen are roughly expected of this LLZO chemical composition. Since the stripped lithium foil completely delaminated during cell disassembly, the SEM/EDS shows a smooth LLZO surface (Supplementary Figure 3e-f). Finally, we note that ^{7}Li CSI images the pristine cell following disassembly, without destructive cross-sectioning of the cell and while keeping the cell in an inert vial; EDS, however, requires destructive cross-sectioning and results in exposure to air for a few seconds during transfer to the SEM/EDS.

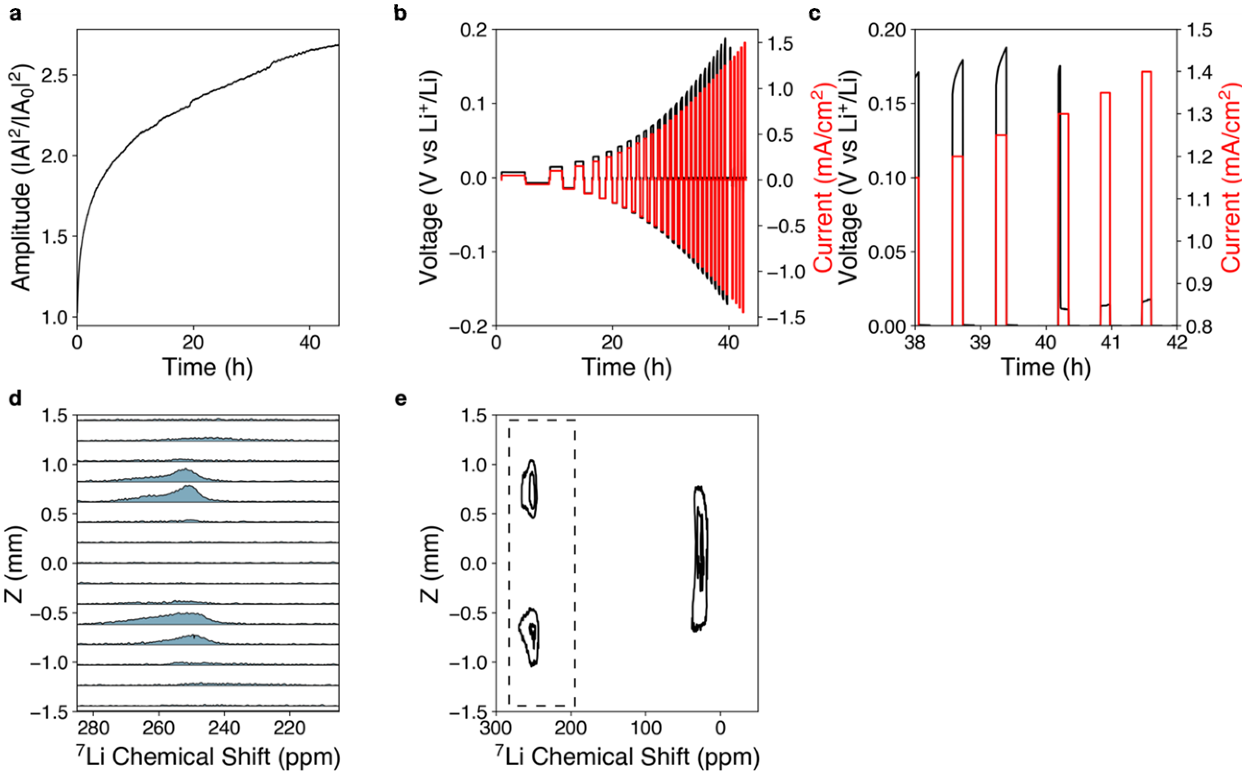

Supplementary Figure 2. Current ramp test for determination of critical current density at 7.4 MPa. Initial current at 0.05 mA/cm^2 with 0.2 mAh charge passed, and increasing current increments of 0.05 mA until short. (a) Normalized acoustic amplitude, with slow increase due to lithium foil creep from high stack pressure (decreased foil thickness resulting in less material for acoustic transmission). (b) Voltage curves of current ramp test. (c) Zoomed in view of electrochemical short at around t = 40 h (~1.3 mA/cm^2). (d) 1D ^{7}Li NMR spectra as a function of z-position taken from ^{7}Li MRI (e). (e) ^{7}Li chemical shift image (CSI) contour plot showing two lithium metal peaks corresponding to the two lithium metal foils and minimal presence of microstructure. The acoustic amplitude is affected by plastic deformation of lithium foil, which decreases the thickness through which the acoustic wave propagates, increasing the amplitude intensity. The amplitude is not sensitive to the sudden and localized electrochemical short at t = 40 h.

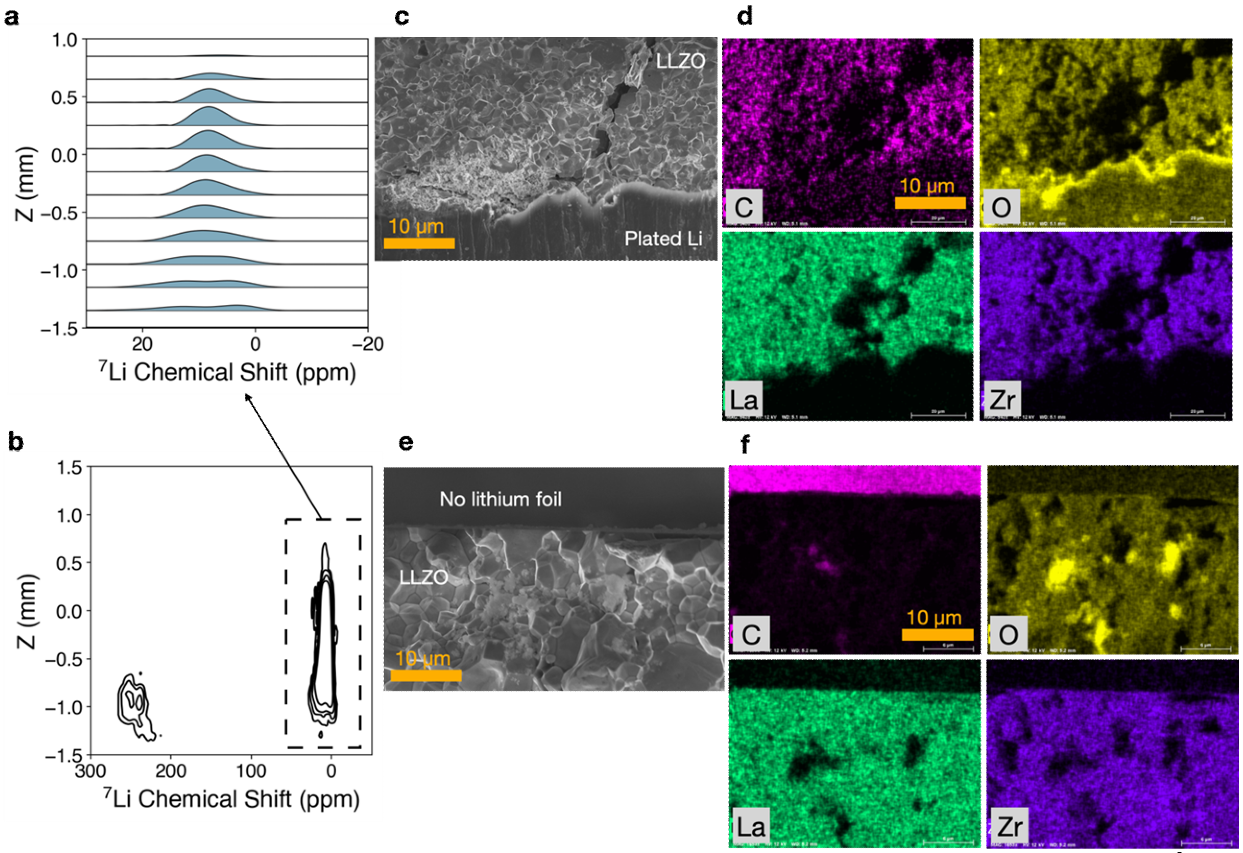

Supplementary Figure 3. Chemical analysis of cell depicted in Figure 2 (2 MPa, 0.2 mA/cm^2). (a) 1D ^{7}Li NMR spectra as a function of z-position in ex situ ^{7}Li CSI (b). Spectra are zoomed in to ~0 ppm to highlight signal arising from LLZO electrolyte. Spectra are approximately 200 µm apart which indicates the spatial resolution of the ^{7}Li MRI image. (b) Ex situ ^{7}Li CSI contour plot of cycled Li/LLZO cell, with lithium metal signal shown at ~240 ppm and referenced to LLZO at 2.3 ppm. (c) SEM cross-section of the plated lithium – LLZO side, indicating an intact interface without presence of voids, but an increase in the interfacial roughness due to uneven plating. (d) Corresponding EDS of the area shown in (c). (e) SEM cross-section of the stripped lithium metal – LLZO interface, where the stripped lithium foil has fallen off during cell disassembly due to poor contact. (f) Energy dispersive X-ray spectroscopy (EDS) of the area shown in (e), indicating presence of lanthanum, zirconium, and oxygen in expected ratios corresponding to $Li_7La_3Zr_2O_{12}$, and adventitious carbon species. Areas on LLZO cross-section with high oxygen concentration correspond to areas of possible lithium deposits as shown in the SEM.

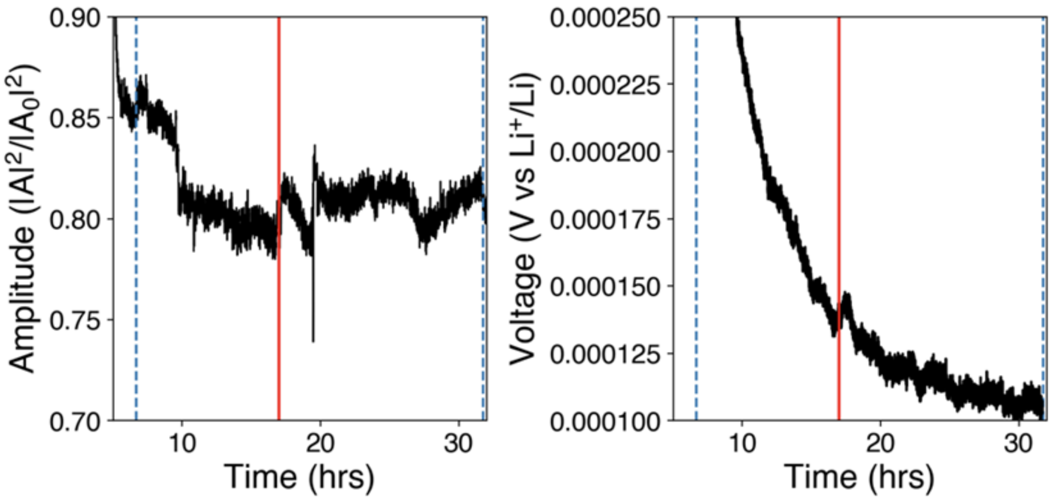

Supplementary Figure 4. Zoomed in view (between t = 5 h and t = 32 h) of acoustic amplitude and voltage profile during 25 h OCV step for cell at 2 MPa stack pressure, shown in Figure 2. Slight perturbations in acoustic amplitude such as at t = 17 h (red line) are correlated with slight changes in the OCV on the order of 10 to 20 μV, which may be caused by mechanical relaxation and movement of lithium at the interface changing the electrochemically active contact area.

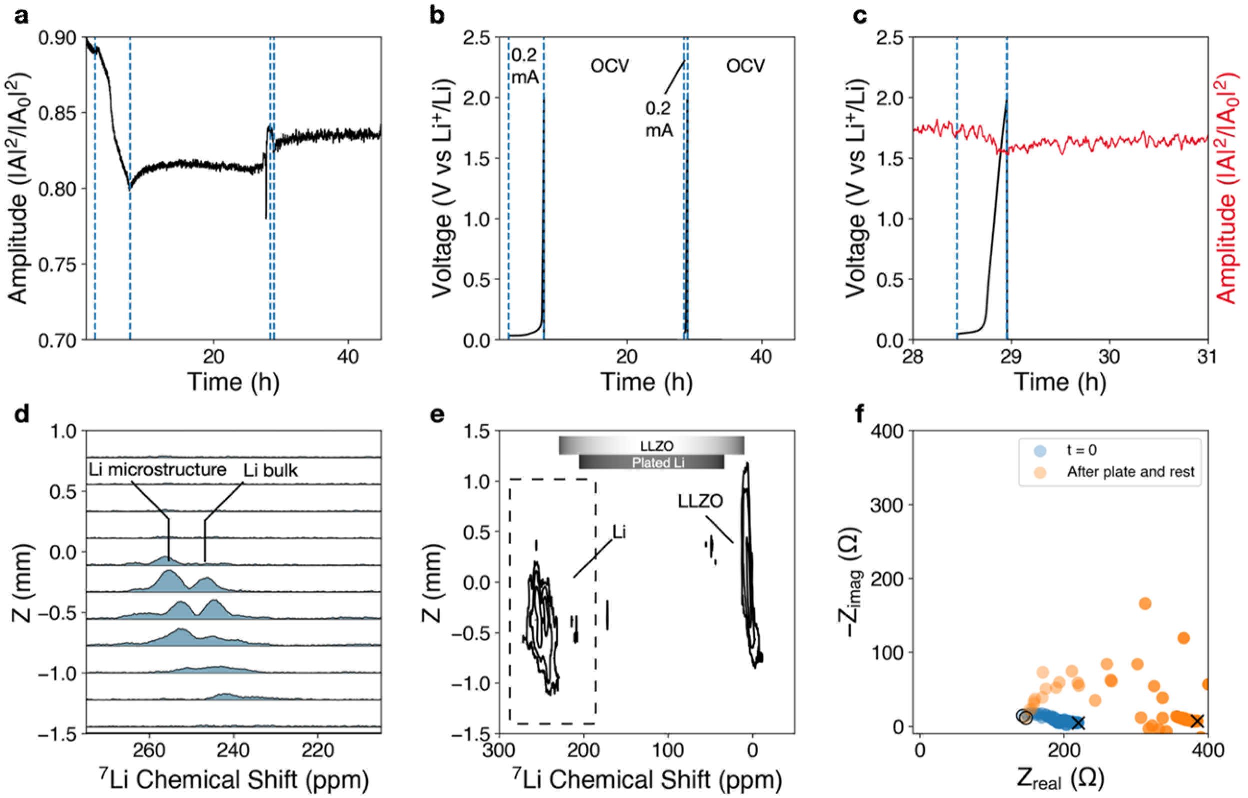

Supplementary Figure 5. Replicate of 2 MPa stack pressure test, polarized to 2 V, showing (a) acoustic attenuation upon applying 0.2 mA/cm^2. (b) A 25 h rest at this stack pressure results in minimal amplitude recovery, with some impedance recovery, but a rapid second polarization step. (c) Zoomed in view of second polarization step (t = 28 h to t = 31 h). (d) 1D ^{7}Li NMR spectra as a function of z-position in ex situ ^{7}Li MRI (e). (e) Ex situ ^{7}Li CSI contour plot of cycled Li/LLZO

cell, with lithium metal signal shown at ~240 ppm and referenced to LLZO at 2.3 ppm. (f) Nyquist plot during initial OCV and after the polarization and rest steps. Open circle indicates high frequency point (7 MHz) and the cross marks the low frequency end point (0.5 Hz).

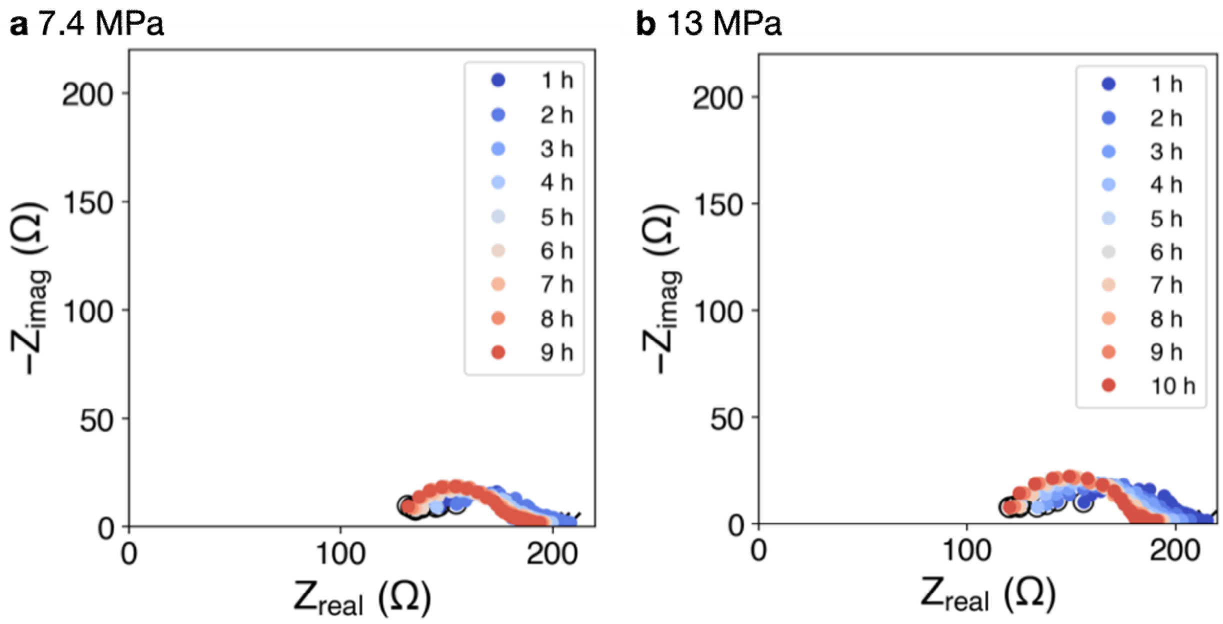

Supplementary Figure 6. Example of hourly EIS tests during initial cell equilibration at OCV, for cells at (a) 7.4 MPa and (b) 13 MPa. At both stack pressures, cell impedance ($R_{CT} + R_{\Omega}$) decreases each hour due to lateral creep of the lithium foil resulting in an increase in Li (s) – LLZO contact area (see Figure 3 and Figure 4).

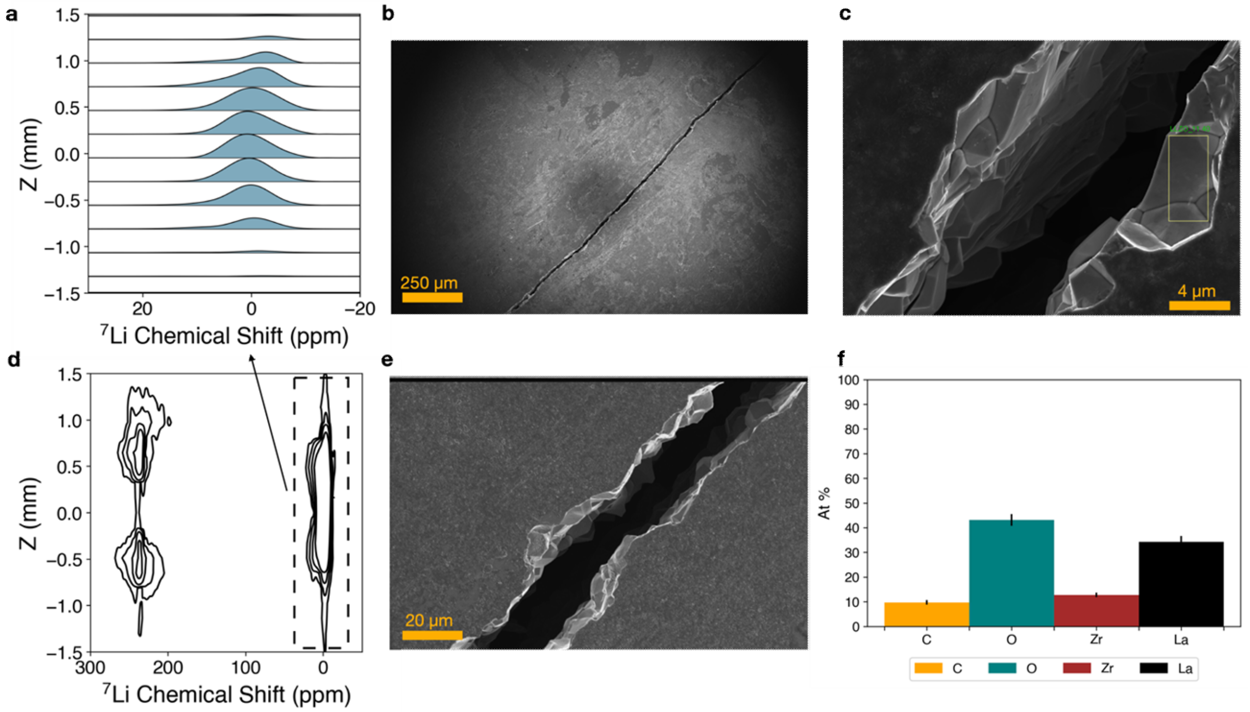

Supplementary Figure 7. (a) 1D ^{7}Li NMR spectra as a function of z-position in ex situ ^{7}Li CSI of the cell presented in Figure 3 of the main text. (b) Low resolution SEM image of crack through center of electrolyte on stripping side, (c) high resolution SEM of crack, with box indicating EDS scan taken, (d) Corresponding ex situ ^{7}Li CSI, (e) high resolution SEM of crack, (f) EDS results for area in (c), confirming primary presence of La, Zr and O without any impurities.

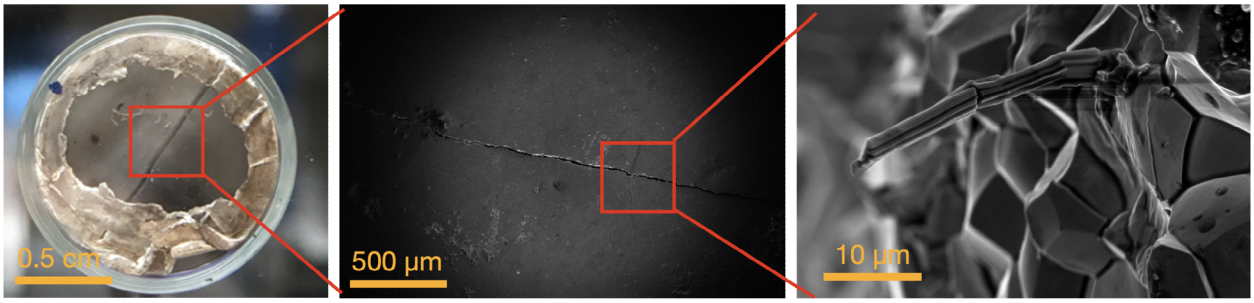

Supplementary Figure 8. Large crack visible through center of LLZO (stripped lithium side). SEM images are conducted at the crack and within the crack. Lithium protrusions out of the polycrystalline LLZO are observed for the cell at 13 MPa, oriented parallel to the electrode (they are growing out of the LLZO polycrystalline sides within the crack).

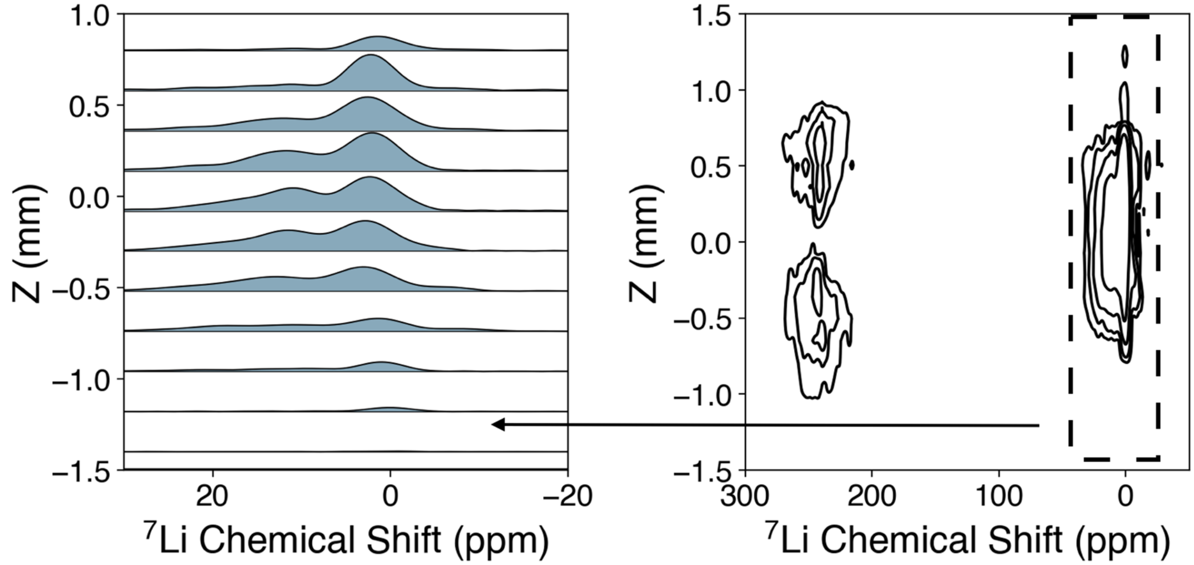

Supplementary Figure 9. Left: 1D ^{7}Li NMR spectra as a function of z-position in ex situ ^{7}Li CSI of the cell presented in Figure 4 of the main text. Right: Corresponding ex situ ^{7}Li CSI.

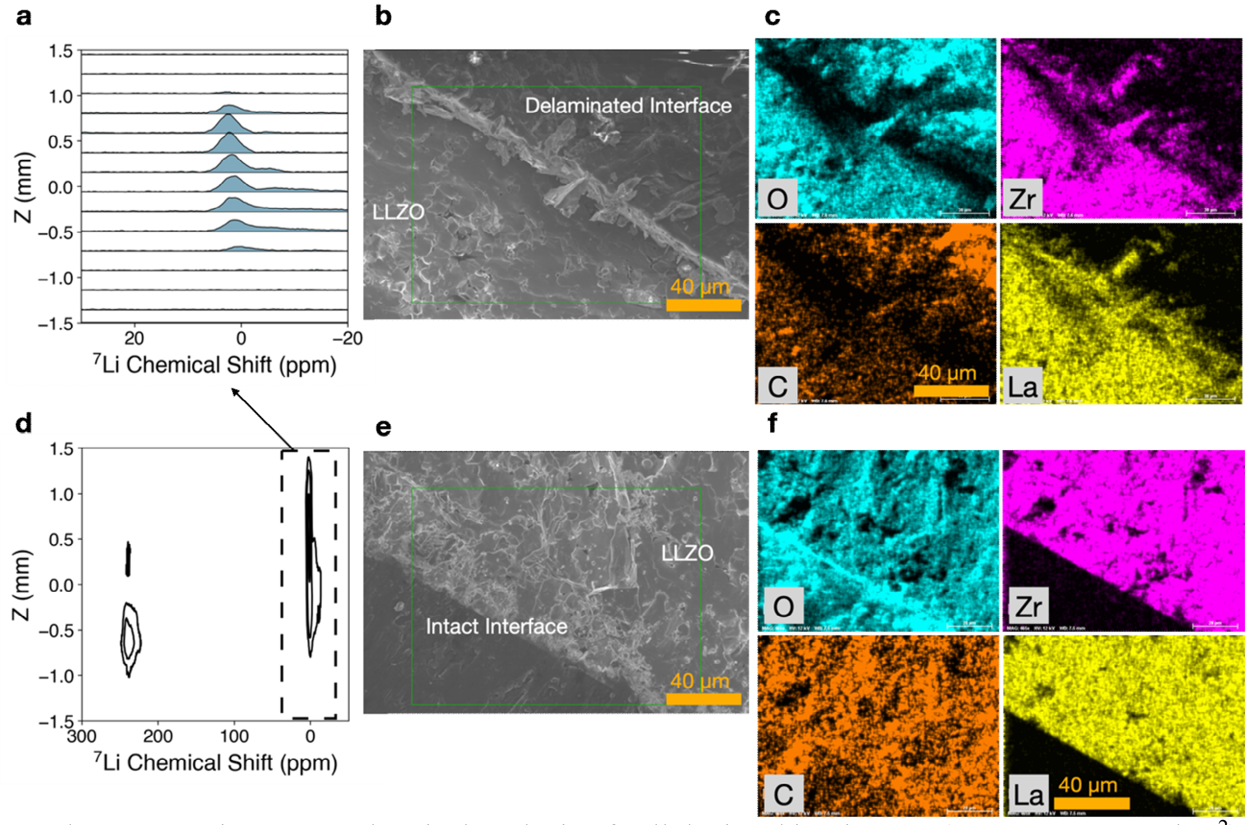

Supplementary Figure 10. Chemical analysis of cell depicted in Figure 5 (7.4 MPa, 0.5 mA/cm^2). (a) 1D ^{7}Li NMR spectra as a function of z-position in ex situ ^{7}Li CSI (Figure d). Spectra are approximately 150 μm apart which indicates the spatial resolution of the ^{7}Li CSI image. (b) SEM cross-section of the stripped lithium metal – LLZO interface, where the stripped lithium foil has fallen off during cell disassembly due to poor contact. (c) Energy dispersive X-ray spectroscopy

(EDS) of the area shown in (b), indicating presence of lanthanum, zirconium, and oxygen in expected ratios corresponding to $Li_7La_3Zr_2O_{12}$, and adventitious carbon species with an uneven electrolyte surface. (d) Ex situ ^{7}Li CSI contour plot of the lithium microstructural chemical shift (~250 ppm) and the LLZO chemical shift (0 ppm). (e) SEM cross-section of the plated lithium – LLZO side, indicating an intact interface without presence of voids, (f) corresponding EDS of the area shown in (e).

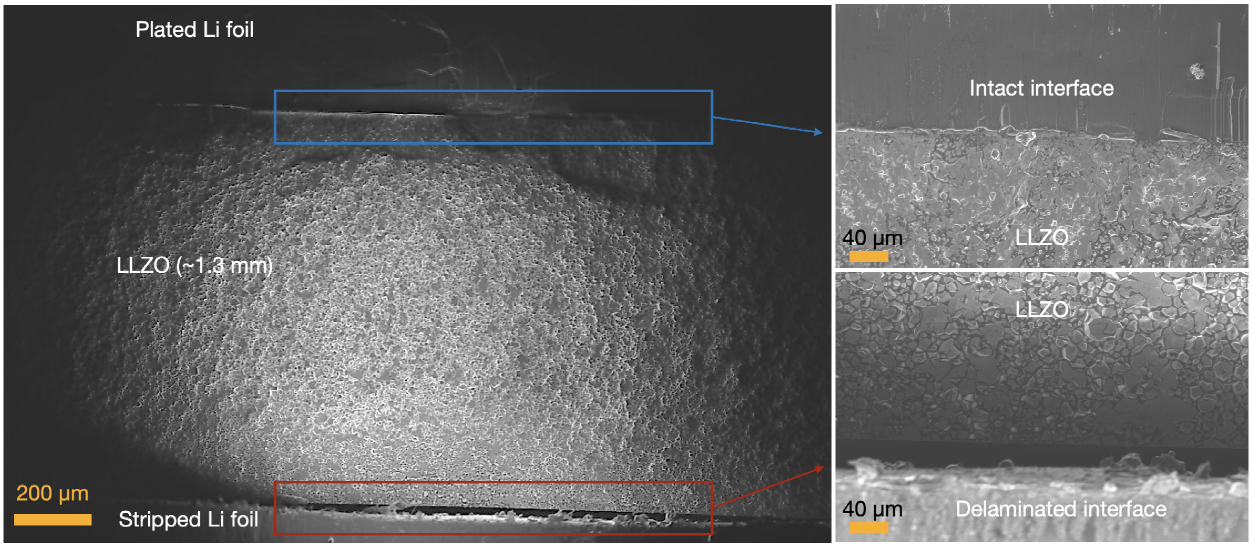

Supplementary Figure 11. Cross-sectional SEM images, showing the plated lithium foil on top, LLZO in the middle, and stripped lithium foil on the bottom. The plated interface is intact, whereas the stripped interface shows delamination, due to loss of contact from void formation. This was a lithium – LLZO – lithium cell galvanostatically polarized at 0.5 mA/cm^2 to 5 V inside the NMR probe.

Supplementary References

1. Majasan, J. *et al.* Recent advances in acoustic diagnostics for electrochemical power systems. *J. Phys. Energy* **3**, 032011 (2021)

2. Villevieille, C., Boinet, M. & Monconduit, L. Direct evidence of morphological changes in conversion type electrodes in Li-ion battery by acoustic emission. *Electrochem. commun.* **12**, 1336–1339 (2010).

3. Gold, L. *et al.* Probing lithium-ion batteries' state-of-charge using ultrasonic transmission--Concept and laboratory testing. *J. Power Sources* **343**, 536–544 (2017).

4. Hsieh, A. G. *et al.* Environmental Science Electrochemical-acoustic time of flight : in operando correlation of physical dynamics with battery charge and health †. *Energy Environ. Sci.* **8**, 1569–1577 (2015).

5. Chang, W. *et al.* Measuring effective stiffness of Li-ion batteries via acoustic signal processing. *J. Mater. Chem. A Mater. Energy Sustain.* **8**, 16624–16635 (2020).

6. Davies, G. *et al.* State of charge and state of health estimation using electrochemical acoustic time of flight analysis. *J. Electrochem. Soc.* **164**, A2746–A2755 (2017).

7. Biwa, S., Nakajima, S. & Ohno, N. On the Acoustic Nonlinearity of Solid-Solid Contact With Pressure-Dependent Interface Stiffness. *J. Appl. Mech.* **71**, 508–515 (2004).
